# Isolation, Genomics-Based and Biochemical Characterization of Bacteriocinogenic Bacteria and Their Bacteriocins, Sourced from the Gastrointestinal Tract of Meat-Producing Pigs

**DOI:** 10.3390/ijms252212210

**Published:** 2024-11-14

**Authors:** Ester Sevillano, Irene Lafuente, Nuria Peña, Luis M. Cintas, Estefanía Muñoz-Atienza, Pablo E. Hernández, Juan Borrero

**Affiliations:** Departamento de Nutrición y Ciencia de los Alimentos (NUTRYCIAL), Sección Departamental de Nutrición y Ciencia de los Alimentos (SD-NUTRYCIAL), Facultad de Veterinaria, Universidad Complutense de Madrid (UCM), Avenida Puerta de Hierro, s/n, 28040 Madrid, Spain; estsev01@ucm.es (E.S.); irelafue@ucm.es (I.L.); nuriapen@ucm.es (N.P.); lcintas@vet.ucm.es (L.M.C.); ehernan@vet.ucm.es (P.E.H.); jborrero@ucm.es (J.B.)

**Keywords:** bacteriocin-producing bacteria, bacteriocins, AMR, whole genome sequencing (WGS), bacteriocin gene clusters (BGC), in vitro cell-free protein synthesis (IV-CFPS), IV-CFPS/SIML, MALDI-TOF MS, LC-MS-MS

## Abstract

Antimicrobial resistance (AMR) poses a significant challenge to animal production due to the widespread use of antibiotics. Therefore, there is an urgent need for alternative antimicrobial strategies to effectively manage bacterial infections, protect animal health, and reduce reliance on antibiotics. This study evaluated the use of emerging approaches and procedures for the isolation, identification, and characterization of bacteriocin-producing bacteria and their bacteriocins, sourced from the gastrointestinal tract (GIT) of meat-producing pigs. Out of 2056 isolates screened against Gram-positive and Gram-negative indicator strains, 20 of the most active antimicrobial isolates were subjected to whole genome sequencing (WGS) for the prediction of coding DNA sequences (CDS) and the identification of bacteriocin gene clusters (BGC) and their functions. The use of an in vitro cell-free protein synthesis (IV-CFPS) protocol and the design of an IV-CFPS coupled to a split-intein mediated ligation (IV-CFPS/SIML) procedure made possible the evaluation of the production and antimicrobial activity of described and putatively novel bacteriocins. A colony MALDI-TOF MS procedure assisted in the identification of class I, II, and III lanthipeptides. MALDI-TOF MS and a targeted proteomics, combined with a massive peptide analysis (LC-MS/MS) approach, has proven valuable for the identification and biochemical characterization of previously described and novel bacteriocins encoded by the isolated bacteriocin-producing strains.

## 1. Introduction

The overuse of antibiotics in animal production for therapeutic, prophylactic, and growth-promoting purposes has led to an increase in antibiotic-resistant bacterial pathogens. This exposure to sublethal doses of antibiotics raises concerns about their transmission to humans and their negative impact on human health [1]. In 2000, the World Health Organization (WHO) highlighted antibiotic resistance as a significant global public health issue and advocated for the gradual elimination of antibiotics as feed additives, particularly those essential for treating human infections. To promote responsible antibiotic use, alternative strategies, such as employing bacteriocins and administering probiotics, are being explored [2,3]. Bacteriocin-producing bacteria and their bacteriocins represent promising alternatives to conventional antibiotics, given their ability to control both animal and foodborne pathogens, including strains resistant to multiple antibiotics [4,5,6]. Bacteriocins spare beneficial microbiota, so they may provide a targeted approach to disease control with minimal impact on the environment and non-target organisms [4,7].

Bacteriocins of Gram-negative bacteria are divided into four main classes: colicins, colicin-like, phage-tail-like bacteriocins, and microcins. Colicins are high molecular weight (20–80 kDa) bactericidal proteins, synthesized by *Escherichia coli* strains under stress conditions. Depending on their mode of action on the target cell, colicins are categorized into three main groups: pore forming colicins (such as colicin A, E1, K, N, S4, Ia, Ib, 10, and others), nuclease colicins (E2–E9), and those that inhibit peptidoglycan synthesis (colicin M). Microcins, conversely, are low-molecular weight (<10 kDa) peptides that play a role in competitive interactions among members of the *Enterobacteriaceae* family. Class I microcins include peptides under 5 kDa (microcins B-17, C7–C51, and J25) that feature complex post-translational modifications. Class II microcins consist of larger peptides (5–10 kDa) without post-translational modifications (including microcins L, V, and 24), as well as linear microcins that carry a C-terminal siderophore (microcins E492, M, H47, I47, and G47). Microcins exert their effects forming pores and disrupting the target membrane [5,8,9].

Most Gram-positive bacteriocins are synthesized as inactive precursors containing a signal peptide or leader sequence. This extension is removed during the export process, yielding a mature and active peptide. Historically, bacteriocins were typically classified in two groups: class I bacteriocins or lanthipeptides, which contain amino acids that undergo post-translational modifications, and class II bacteriocins, characterized by unmodified amino acid residues [10]. However, the current classification of class I now encompasses all ribosomally synthesized peptides that are post-translationally modified (RiPPs) through enzymatic processes during their biosynthesis (e.g., lanthipeptides, head-to-tail cyclized peptides, linear azol(in)-containing peptides, thiopeptides, lasso peptides, sactipeptides, and others). Five classes of lanthipeptides (I to V) have been reported, which differ in the biosynthetic enzymes involved in the formation of methyllanthionine (Me)Lan. Class II bacteriocins are further divided into four subgroups: class IIa (pediocin-like bacteriocins); class IIb (two-peptide bacteriocins); class IIc (leaderless bacteriocins); and class IId (non-pediocin-like, single-peptide bacteriocins). More recently, a third group, class III bacteriocins, has been proposed, encompassing unmodified bacteriocins larger than 10 kDa that function via either bacteriolytic or non-lytic mechanisms of action [11,12,13].

This study aimed to evaluate emerging approaches for the isolation, identification, biochemical characterization, and genetic profiling of bacteriocin-producing bacteria and their bacteriocins. The utilization of commercial bacterial replicators may facilitate the expansion and growth of isolated colonies, while whole genome sequencing (WGS) of the antimicrobial isolates may enable the prediction of their coding DNA sequences (CDS) and the identification of putative bacteriocin gene clusters (BGC) and their functions. Furthermore, the use of an in vitro cell-free protein synthesis (IV-CFPS) protocol and the design of an IV-CFPS coupled to a split-intein mediated ligation (IV-CFPS/SIML) procedure, may help in the evaluation of the production and antimicrobial activity of a number of class IIb, class IIc, class IId, and putative class I circular bacteriocins. Additionally, a colony MALDI-TOF mass spectrometry (MS) procedure may assist in the identification of class I, II, and III lanthipeptides. The combination of advanced mass spectrometry (MS) and LC-MS/MS analysis may prove valuable in the evaluation of different ribosomally synthesized and modified peptides (RiPPs) produced by the bacteriocin-producing strains. This comprehensive analytical strategy may fill a critical gap by integrating synthetic biology techniques and multi-omic methods with bacteriocin research, thereby advancing our understanding of the biological roles and potential biotechnological applications of bacteriocin-producing bacteria and their bacteriocins.

## 2. Results

### 2.1. Isolation of Bacterial Strains and Identification of the Most Active Isolates

A total of 2056 isolates were sourced from samples collected from the contents of the terminal section of the rectum (PG) or the small intestine (SI), cecum (CE), and colon (CO) of meat-producing pigs processed at two different slaughterhouses. Approximately 255 isolates (12.4%) showed direct antimicrobial activity against at least one of the evaluated indicators, as determined by a spot on agar test (SOAT). The indicator strains included various porcine-origin strains, along with two microorganisms, *E. coli* DH5α and *Pediococcus damnosus* CECT 4797, commonly used in our group due to their higher sensitivity to a wide range of known bacteriocins (Table S1). From these isolates, 19 Gram-negative and 23 Gram-positive isolates were further selected for determination of their RAPD-PCR patterns to identify a single candidate from those sharing the same pattern.

Among the 19 most active and diverse Gram-negative isolates, seven of them were selected based on RAPD-PCR results and identified through 16S rDNA sequencing as one strain of *Pseudomonas alcaligenes* (PG7) and six strains of *E. coli* (PG9, PG14, PG15, PG18, P8CEA3, and P8COA2). *P. alcaligenes* PG7 and four out of the six *E. coli* isolates displayed antimicrobial activity against at least one of the *E. coli* indicator strains. *E. coli* PG15 was only active against *Salmonella* Choleraesuis (*S. enterica* subsp. *enterica* serovar Choleraesuis), while *E. coli* P8COA2 was active against most *E. coli* indicators and *Salmonella paratyphi* CECT 554 (Table 1).

Thirteen out of the most active and diverse Gram-positive isolates were selected and identified by 16S rDNA sequencing as follows: *Limosilactobacillus reuteri* (P1CEA2 and P8SIA3); *Ligilactobacillus salivarius* (P1CEA3 and PG21); *Lactobacillus johnsonii* (P8CEA12, P8COA6, and P8COA7); *Paenibacillus dendritiformis* (P1CEA1); *Paenibacillus lentus* (P8CEA4, P8CEA5, and P8SIA1); *Staphylococcus saprophyticus* P1CEA4; and *Staphylococcus simulans* P8CEA7. Eight of these 13 Gram-positive isolates were active against all the indicator strains tested. *L. reuteri* P1CEA2 exhibited activity only against *P. damnosus* CECT 4797, while *P. lentus* P8CEA4, P8CEA5, and P8SIA1 showed antimicrobial activity against all tested indicator strains except *P. damnosus* CECT 4797. *S. simulans* P8CEA7 was active against all evaluated indicator strains except *S. aureus* ZTA11/00117ST and *S. aureus* ZTA11/00310ST (Table 2). None of the identified isolates showed hemolytic or gelatinase activities.

### 2.2. Whole Genome Sequencing (WGS), Bioinformatic Identification of Bacteriocins and In Vitro Cell-Free Protein Synthesis (IV-CFPS) of Bacteriocins Encoded by Selected Gram-Negative Producers

The WGS of the seven selected Gram-negative isolates allowed for their assembly into contigs, enabling the prediction of their coding DNA sequences (CDS). The KmerFinder webserver confirmed the species identification from the 16S rDNA PCR results. Analysis using bacteriocin mining tools, BAGEL v.4.0 and antiSMASH, identified various bacteriocin gene clusters (BGC) in their genomes. Notably, *P. alcaligenes* PG7 was found to encode a putative S-type pyocin (Table 3 and Table S3 and Figure S1), but IV-CFPS production of this pyocin did not yield detectable antimicrobial activity against any of the tested indicator strains, including *E. coli* DH5α, *P. fluorescens* B52, and *P. putida* 3.

Additionally, *E. coli* PG9 also encoded a hypothetical unnamed bacteriocin like-peptide (BLP) and colicins E6, Ia, and E1. *E. coli* PG14, and *E. coli* PG15, while exhibiting different antimicrobial activities, shared the same microcins H47, M, and V, and colicin Ia in their genomes. *E. coli* PG18 encoded colicin Ib, while *E. coli* P8CEA3 encoded microcins V and B17 and colicin E1. *E. coli* P8COA2 encoded the same hypothetical BLP as *E. coli* PG9 and the colicins Ib, S4, 10, and E2. Given that most *E. coli* isolates encoded previously described bacteriocins, no further evaluation of IV-CFPS production or determination of antimicrobial activity was conducted for the identified bacteriocins in these active *E. coli* isolates (Table 3 and Table S3 and Figure S1).

**Figure 1:**
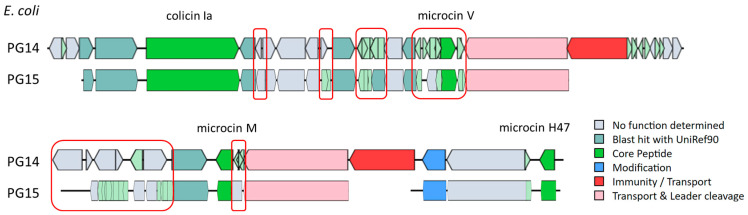
Alignment of the bacteriocin gene clusters (BGC) from *E. coli* PG14 and *E. coli* PG15 isolates, which encode identical bacteriocins as performed using BAGELv.4.0. ORFs are represented by arrows and those with a predicted function are indicated by gene identity and/or color. Red squares highlight regions where gene rearrangements have been identified.

The genomes of *E. coli* PG14 and *E. coli* PG15, sharing identical bacteriocins but different spectra of antimicrobial activity, were analyzed using Mauve Multiple Genome Alignment. The results indicated that the *E. coli* PG14 genome contained a non-aligned region, likely harboring sequence elements specific to this particular strain (Figure S2A). Additionally, a comparison of the BCG sequences obtained from BAGEL v.4.0, related to the synthesis of the bacteriocins colicin Ia and microcins V, M and H47 by *E. coli* PG14 and *E. coli* PG15, revealed significant differences in the sequences of the genes adjacent to the structural bacteriocin genes (Figure 1).

### 2.3. Whole Genome Sequencing (WGS) and Bioinformatic Identification of Bacteriocins Encoded by Selected Gram-Positive Producers

The WGS of the thirteen Gram-positive isolates allowed for the assembly of contigs, prediction of their CDS, and confirmation of species identification. Analysis using the BAGEL v.4.0 and antiSMASH web tools facilitated the bioinformatic analysis of their bacteriocins. Although *L. reuteri* P1CEA2 and P8SIA3 demonstrated antimicrobial activity, they did not encode any predicted bacteriocins in their genomes. In contrast, the *L. salivarius* P1CEA3 strain encoded two BGC for the class IId salivaricin B and class IIb Abp118 (α+β), as well as an additional BGC for the class I lanthipeptide nisin S [14,15] (Table 4 and Figure S3). The *L. salivarius* PG21 genome encoded the class IId bactofencin A and a BGC containing four bacteriocins, three of which were predicted to be class IIb two-component bacteriocins: salivaricin T (α+β) like peptide (LP), plantaricin NC8 (α+β) LP, and plantaricin S (α+β) LP, along with the class IId gassericin T LP/lactacin F LafA LP (Table 4, Figures S3 and S4). The three *L. johnsonii* P8CEA12, P8COA6, and P8COA7 strains exhibited similar yet distinct antimicrobial activities, while their genomes encoded the identical class III bacteriocin helveticin J LP (Table 4 and Figure S3).

The *P. dendritiformis* P1CEA1 strain encoded a predicted class I proteusin LP from the NHLP leader peptide family of RiPP precursors, as well as the lasso peptide paeninodin, a bacteriocin from the heterocycloanthracin/sonorensin family (thiazole/oxazole-modified microcin (TOMM)), and a class I lanthipeptide from the FDLD family known as paenicidin LP (Table 4 and Figure S3). The *P. lentus* P8CEA4, P8CEA5, and P8SIA1 strains contained BGC for a proteusin LP precursor, a class III lanthipeptide LP, the sactipeptide thuricin 17 LP, a class IId bacteriocin thermophilin A LP, and two putative class I circular bacteriocins related to the circularin A/uberolysin family, designated as circular bacteriocins PL1 and PL2 (Table 4, Figures S3 and S5). The *S. saprophyticus* P1CEA4 strain encoded a BGC predicted to produce the class IIc epidermicin LP (Table 4, Figures S3 and S4). Lastly, the *S. simulans* P8CEA7 strain encoded a class IId lactococcin 972 LP family bacteriocin and a putative class II lanthipeptide (α+β) (Table 4 and Figure S3).

The evaluation of the *L. johnsonii* genomes revealed larger disparities in genome similarities between strains P8COA6-P8COA7 and P8COA6-P8CEA12 than between P8COA7-P8CEA12 (Figure S2B). In contrast, the comparative evaluation of the genomes of *P. lentus* P8CEA4, P8CEA5, and P8SIA1 demonstrated an homologous organization (Figure S2C). Consequently, the focus on bacteriocins encoded by the Gram-positive isolates will be narrowed down to eight strains: *L. salivarius* P1CEA3, *L. salivarius* PG21, *L. johnsonii* P8CEA12, *L. johnsonii* P8COA6, *P. dendritiformis* P1CEA1, *P. lentus* P8CEA5, *S. saprophyticus* P1CEA4, and *S. simulans* P8CEA7.

### 2.4. In Vitro Cell-Free Protein Synthesis (IV-CFPS) of Bacteriocins Encoded by Selected Gram-Positive Strains and Evaluation of Their Antimicrobial Activity

**Figure 2:**
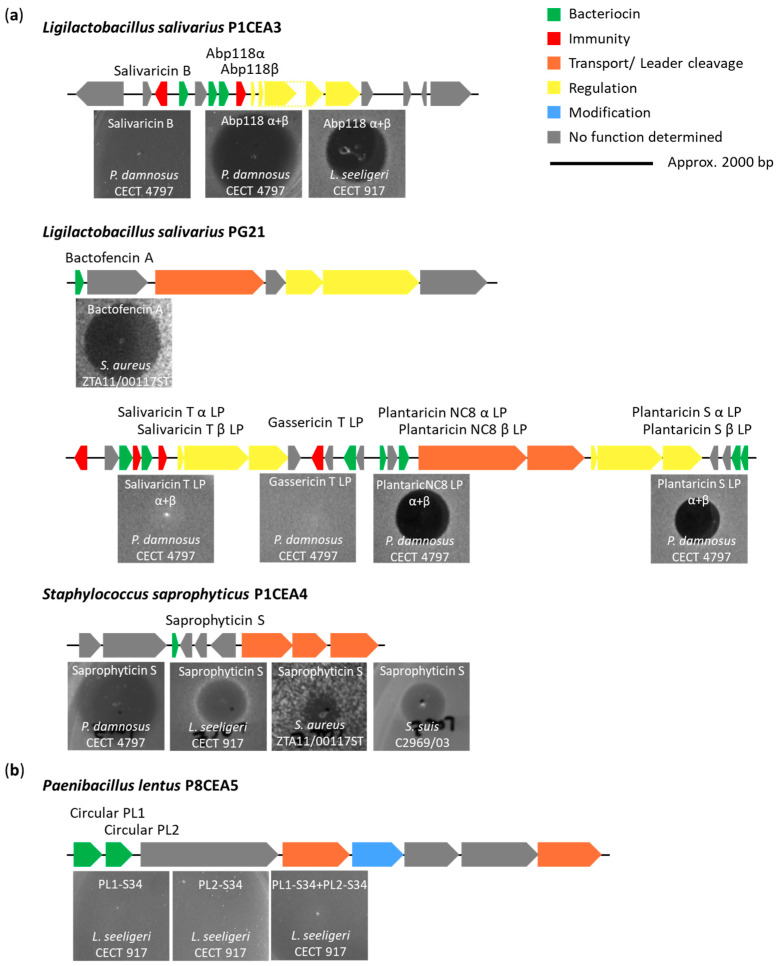
(**a**). Bacteriocin gene cluster (BGC) and antimicrobial activity assessed by the spot-on-agar test (SOAT) of IV-CFPS-produced class II bacteriocins from *L. salivarius* P1CEA3, *L. salivarius* PG21, and *S. saprophyticus* P1CEA4 against different indicator strains. (**b**). BGC and antimicrobial activity evaluated by the spot-on-agar test (SOAT) of IV-CFPS/SIML-produced putative class I circular bacteriocins PL1 and PL2, encoded by *P. lentus* P8CEA5, against different indicator strains. ORFs are represented by arrows and those with a predicted function are indicated by gene identity and/or color.

Total genomic DNA from the Gram-positive strains selected was used as template for amplification by PCR of genes encoding the mature class II bacteriocins, which were synthesized in vitro (IV-CFPS) using specific primers (Table S2). In this study, the IV-CFPS production of the encoded salivaricin B by *L. salivarius* P1CEA3 showed no antimicrobial activity against *P. damnosus* CECT 4797. However, the bacteriocin Abp118 demonstrated antimicrobial activity against *P. damnosus* CECT 4797 and *L. seeligeri* CECT 917 when both peptides (α+β) were mixed (Figure 2a). The chemically synthesized bactofencin A, encoded by *L. salivarius* PG21, was active against *S. aureus* ZTA11/00117ST, while the bacteriocins gassericin T LP, lactacin F LafA LP, and salivaricin T (α+β) LP did not demonstrate antimicrobial activity against any of the tested indicator strains. However, the bacteriocins plantaricin NC8 (α+β) LP and plantaricin S (α+β) LP, also encoded by *L. salivarius* PG21, showed antimicrobial activity against *P. damnosus* CECT 4797 when both peptides (α+β) were mixed (Figure 2a).

The saprophyticin S encoded by *S. saprophyticus* P1CEA4 exhibited antimicrobial activity against all tested indicator strains, including *P. damnosus* CECT 4797, *L. seeligeri* CECT 917, *S. aureus* ZTA11/00117ST, and *S. suis* C2969/03 (Figure 2a). However, no efforts were made to evaluate the IV-CFPS production or antimicrobial activity of the lactococcin 972 LP encoded by *S. simulans* P8CEA7.

The putative circular bacteriocins PL1 and PL2, encoded by *P. lentus* P8CEA5, were evaluated for their production and antimicrobial activity using a specifically designed IV-CFPS/SIML procedure for the ligation of circular bacteriocins (Figure S5). However, none of the tested reactions showed antimicrobial activity against any of the indicator strains, including *P. damnosus* CECT 4797, *L. seeligeri* CECT 917, *B. cereus* ICM17/00252, *B. pumilus* PE12, *B. toyonensis* NM11, and *P. dendritiformis* P1CEA1 (Figure 2b).

### 2.5. Colony MALDI-TOF MS Analysis of Bacteriocins Encoded by Selected Gram-Positive Strains

**Figure 3:**
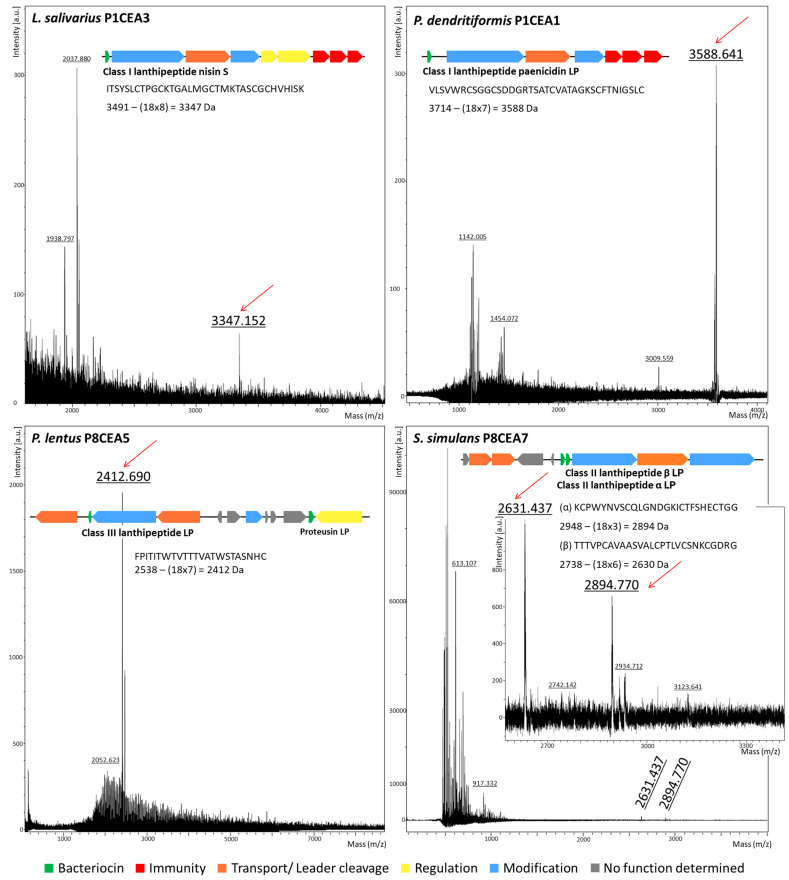
Colony MALDI-TOF MS analysis of samples from *L. salivarius* P1CEA3, *P. dendritiformis* P1CEA1, *P. lentus* P8CEA5, and *S. simulans* P8CEA5, which encode different class I, II, and III lanthipeptide bacteriocins. The BGC and amino acid sequences of the lanthipeptides are represented. ORFs are represented by arrows and those with a predicted function are indicated by gene identity and/or color.

All Gram-positive bacterial strains, except *L. johnsonii* P8CEA12 and P8COA6, which encode the bacteriocin helveticin J LP, were subjected to colony MALDI-TOF MS analysis. The combination of *L. salivarius* P1CEA3 with isopropanol containing TFA, followed by analysis using colony MALDI-TOF MS, revealed a peptide peak with an observed molecular mass of 3347.15 Da. This corresponds to the calculated molecular mass of the lanthipeptide nisin S (3491 Da) with 8 dehydrated amino acid residues. No peptide signals corresponding to the calculated masses of the bacteriocins salivaricin B, Abp118 α, and Abp118 β, were observed (Figure 3). Similarly, no peptide peaks matching the calculated molecular masses of bactofencin A, gassericin T LP/lactacin F LafA LP, or the two component bacteriocins salivaricin T (α+β) LP, plantaricin NC8 (α+β) LP, and plantaricin S (α+β) LP were identified among the peptides observed from the *L. salivarius* PG21 mix.

However, colony MALDI-TOF MS analysis of the *P. dendritiformis* P1CEA1 samples revealed a peptide fragment with an observed molecular mass of 3588.64 Da, corresponding to the calculated molecular mass of the class I lanthipeptide paenicidin LP (3714 Da) with seven dehydrated residues. No evidence was found for the presence of the proteusin peptide LP or the heterocycloanthracin/sonorensin LP. The colony MALDI-TOF MS analysis of *P. lentus* P8CEA5 samples identified a peptide fragment with an observed molecular mass of 2412.69 Da, corresponding to the calculated molecular mass of the class III lanthipeptide LP (2538 Da) with seven dehydrated residues (Figure 3). Additionally, colony-derived samples from *S. simulans* P8CEA7 revealed peptide peaks corresponding to the class II lanthipeptide (α+β) LP, although lactococcin 972 LP was not observed. It may be hypothesized that the lanthipeptide α, with an observed mass of 2894.77 Da and a calculated mass of 2948 Da (containing three dehydrated residues), may have lost the [CNTSA] amino acid residues at its C-terminal end during processing and transport. The lanthipeptide β showed an observed mass of 2631.43 Da, corresponding to a calculated mass of 2738 Da with six dehydrated residues (Figure 3).

### 2.6. Purification of the Bacteriocins Produced by P. lentus P8CEA5, MALDI-TOF MS Analysis, and LC-MS/MS Evaluation by Targeted Proteomics Combined with Massive Peptide Analysis of the Trypsinized Fractions

**Figure 4:**
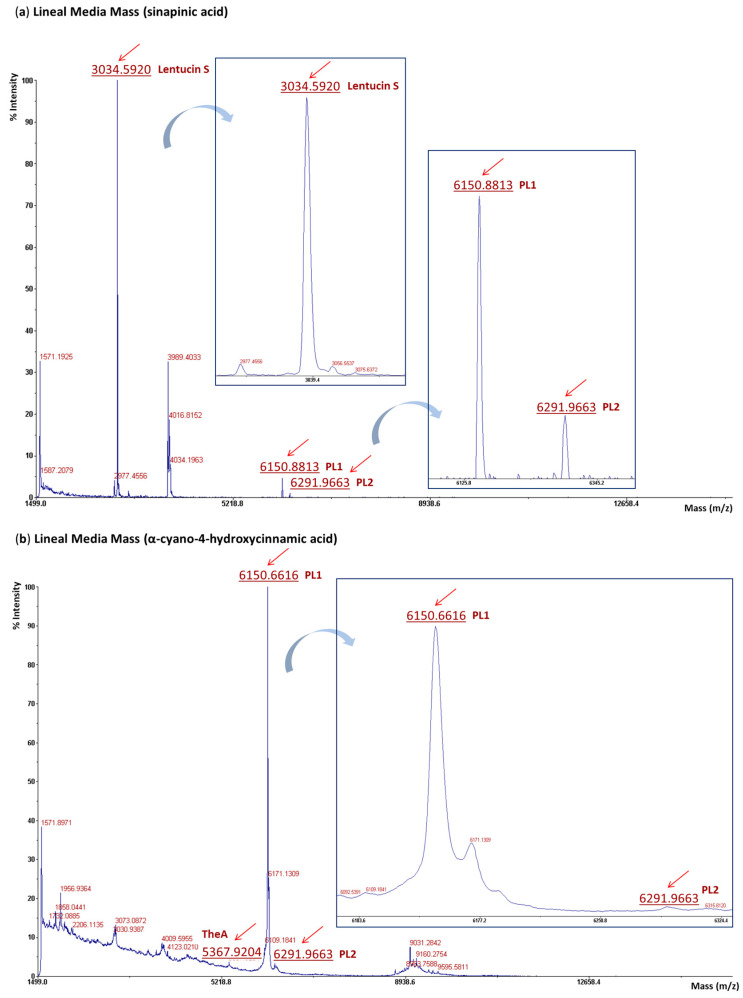
MALDI-TOF MS analysis of the RP-FPLC active fractions from the purified supernatants of *P. lentus* P8CEA5. (**a**). Sinapinic acid matrix, and (**b**). α-cyano-4-hydroxycinnamic acid matrix. Peptide peaks with the molecular masses as observed for the predicted lentucin S (thuricin 17 family bacteriocin), thermophilin A LP (TheA) and the circular bacteriocins PL1 and PL2.

The purification of the CFS of *P. lentus* P8CEA5, grown for 48 h in TSB at 37 °C, involved a multi-chromatographic procedure followed by MALDI-TOF MS analysis of the RP-PFLC active fractions. This analysis revealed a peptide with a mass of 3034.58 Da, two peptide peaks of 6150.86 Da and 6292.44 Da, and a minor peptide fragment of 5367.92 Da (Figure 4). The peptide of 3034.58 Da is 8 Da lower than the calculated molecular mass of the sactipeptide thuricin 17 LP encoded by *P. lentus* P8CEA5, thus suggesting that the produced peptide may be subject to sulfur to α-carbon crosslinks. The observed two peptide peaks of 6150.86 Da and 6292.44 Da, although 18 Da lower than the predicted molecular masses of the putative class I circular bacteriocins PL1 and PL2, may indicate the circularization of these peptides by a head-to-tail peptide bond (−18 Da) between their N- and C-terminal residues, supporting the recognition of circular bacteriocin production by this strain. The observed peptide of 5367.92 Da is 14 Da higher than the calculated molecular mass (5353 Da) of the class IId thermophilin A LP, suggesting potential methylation of an amino acid within the peptide chain and supporting the production of this bacteriocin by *P. lentus* P1CEA5. However, the presence of an unprocessed polytheonamide precursor (proteusin peptide LP, NHLP leader peptide family) in the purified CFS of *P. lentus* P8CEA5 could not be confirmed (Figure 4).

The RP-FPLC active fractions from the purified CFS of *P. lentus* P8CEA5 were digested with trypsin, and the resulting peptide fragments subjected to LC-MS/MS analysis and identification by using a targeted proteomics approach combined with massive peptide analysis. The identification in the active fractions of the peptide fragment [ALTLVSALSGLNTID], which covers 48.4% of the sequence of the sactipeptide thuricin 17 LP, further supports the production of this bacteriocin by *P. lentus* P8CEA5. The peptide fragment [ADSWKDLGK] accounted for 16.6% of the sequence of the class IId thermophilin A LP. No peptide fragments corresponding to the circular bacteriocins PL1 and PL2 were observed.

## 3. Discussion

Antimicrobial resistance (AMR) represent a major challenge in animal production, where the widespread use of antibiotics remains common practice. Consequently, there is a pressing need for alternative antimicrobial approaches to control bacterial infections, ensuring animal health while reducing dependency on antibiotics [4,16,17]. This study aims to explore the use of emerging approaches and procedures for the isolation, identification, and characterization of bacteriocin-producing bacteria and their bacteriocins, sourced from the GIT of meat-producing pigs.

With the aim of identifying bacteriocin-producing bacteria and bacteriocins targeting bacterial species relevant in swine production, a screening study was conducted. Most of the active Gram-negative bacteria were isolated from the rectum (PG) while the majority of active Gram-positive bacteria were sourced from the cecum (CE) of sampled pigs (Table 1 and Table 2). The small intestine (SI) primarily hosts Firmicutes and Proteobacteria, and exhibits a reduced bacterial diversity within the GIT communities. In contrast, the cecum and colon (CO) harbor a variety of genera among which members of the phyla Firmicutes and Bacteroidetes are predominant [18,19].

In this study, all *E. coli* isolates contained BGC for the production of bacteriocins (Table 3, Table S3 and Figure S1). *E. coli* P8COA2 was active against *S. paratyphi* CECT 554, likely due to its encoding of colicin E2, a bateriocin known for its potent DNase activity [20,21]. The *P. alcaligenes* PG7 strain encodes a predicted S-type pyocin, a colicin-like bacteriocin with DNase activity [22,23]. However, the IV-CFPS production of this putative bacteriocin did not show antimicrobial activity against any of the tested indicator strains. This may indicate that the mature sequence of the bacteriocin was not accurately determined, that the predicted S-type pyocin is inactive against the evaluated bacterial indicators, or that this peptide does not qualify as a true bacteriocin.

Regarding the Gram-positive isolates evaluated in this study, no bacteriocins were identified in the genomes of *L. reuteri* P1CEA2 and P8SIA3. Therefore, their antimicrobial activity may stem from the production of organic acids, carbohydrate- and/or fatty acid-derived metabolites, or other yet unidentified secondary metabolites [24,25]. In contrast, the *L. johnsonii* P8CEA12 and P8COA6 strains encoded helveticin J, a class III heat-labile bacteriocin, with a broad-spectrum antimicrobial activity [26,27]. The protein NX371 with a 98,1% homology to helveticin J, produced by *L. acidophilus* NX2-6, has been proposed as a broad-spectrum preservative for the dairy industry [28]. *L. johnsonii* L16, a porcine-derived strain encoding helveticin J, has recently been proposed as a probiotic strain in swine production [29].

The *L. salivarius* P1CEA3 and *L. salivarius* PG21 strains showed distinct antimicrobial activities against different indicator strains (Table 2). The IV-CFPS production of the bacteriocin Abp118 (α+β) showed antimicrobial activity but not that of salivaricin B (SalB) However, previous work determined that neither the SalB nor Abp118 (α+β) peptides were actually produced by the producer cells [15]. The production of nisin S by *L. salivarius* P1CEA3 has been confirmed in the supernatants of the producer cells [15]. This is the first nisin variant identified in *L. salivarius*. Future studies will evaluate the potential of *L. salivarius* P1CEA3 as a probiotic of interest for swine production and other biotechnological applications [14].

In the *L. salivarius* PG21 strain, only the chemically synthesized bactofencin A and the IV-CFPS produced plantaricin NC8 (α+β) LP and plantaricin S (α+β) LP exhibited antimicrobial activity (Figure 2). Bactofencin A is a class IId bacteriocin with an unusual highly cationic N-terminus and a single disulfide bond between C7 and C22, resulting in a prominent terminal loop [30]. Plantaricin NC8 and plantaricin S are previously described class IIb bacteriocins produced by *L. plantarum* [31,32]. However, the encoded salivaricin T (α+β) LP showed no antimicrobial activity after its IV-CFPS-derived production. This lack of activity may be related to the presence of tyrosine instead of cysteine (Y29C) in its amino acid sequence as compared to the salivaricin T produced by *L. salivarius* DPC6488 [33], which could be crucial for the secondary structure of the bacteriocin. Additionally, the predicted gassericin T LP/lactacin F LafA LP was also non-functional after in vitro production. This inactivity might be due to the loss of the last five residues in the C-terminal [GKIRK] compared to native gassericin T (Figure S4), or to the absence of the X-chain, which was not found in the BGC of *L. salivarius* PG21 but is present in the native two-component class IIb bacteriocin (LafA+LafX) [33,34]. Nevertheless, the coding by *L. salivarius* PG21 of multiple bacteriocins of different classes and mode of action may surely lead to a higher antimicrobial potency and broad spectrum of inhibition [35]. Such a bacteriocin diversity may also contribute to the ability of *L. salivarius* PG21 to dominate and compete within the complex microbiota of the porcine GIT.

*Paenibacillus* species are known for producing antimicrobial metabolites, including bacteriocins [36]. In this study, *P. dendritiformis* P1CEA1 showed to held BGC encoding multiple bacteriocins (Table 4 and Figure S3). The presence of a BGC encoding nitrile hydratase-like precursor genes (NHLPs) suggests the potential synthesis of a proteusin LP (NHLP-derived RiPPs), which are linear peptides composed of polytheonamides [37,38]. Conversely, another BGC encodes a potential lasso peptide, a class of RiPPs, characterized by a macrolactam ring formed between the N-terminus and an internal aspartic (D) or glutamic acid (E) residue [39]. Additionally, another BGC appears to encode a heterocycloanthracin/sonorensin LP bacteriocin belonging to the class I subfamily of bacteriocins within the TOMMs (thiazole/oxazole-modified microcins) group. These bacteriocins, such as microcin B17, sonorensin, and lichenicidin, are produced as small precursor peptides that undergo post-translational modifications to incorporate thiazole and (methyl)oxazole heterocycles [40]. However, and most importantly, colony MALDI-TOF MS analysis of *P. dendritiformis* P1CEA3 identified a peptide corresponding to the class I lanthipeptide paenicidin LP with seven dehydrated residues, but not the presence of the proteusin LP, the lasso peptide paeninodin LP, or the sonorensin LP (Figure 3).

All the *P. lentus* P8CEA4, P8CEA5, and P8SIA1 strains exhibited antimicrobial activity against all the indicator strains tested except *P. damnosus* CECT 4797 (Table 2). Thus, these strains were active against relevant pathogens but not against a lactic acid bacteria (LAB) strain sensitive to many bacteriocins. The *Paenibacillus* spp. may have evolved in environments where LAB were not a significant threat, leading to a lack of selective pressure to develop antimicrobial activity against them. The presence of BGC in the genomes of *P. lentus* P8CEA4, P8CEA5, and P8SIA1 highlights their potential for producing multiple bacteriocins (Table 4 and Figure S3). A colony MALDI-TOF MS analysis of *P. lentus* P8CEA5 permitted the identification of a peptide peak corresponding to a class III lanthipeptide LP with seven dehydrated residues (Figure 3).

The production of the class III lanthipeptide by *P. lentus* P8CEA5 prompted us to investigate the presence of other bacteriocins in the CFS of this strain. Therefore, the CFS from appropriately grown cells of *P. lentus* P8CEA5 was purified using a multi-chromatographic procedure, and the resulting RP-FPLC-derived antimicrobial fractions analyzed by MALDI-TOF MS. The results revealed the presence of a putative class I sactipeptide similar to thuricin 17/thurincin H [41,42], with a molecular mass of 3034.58 Da (Figure 4). Class I sactipeptides are RiPPs characterized by an intramolecular sulfur-to-α-carbon thioether bond, known as sactionine, which is formed by a radical S-adenosylmethionine (rSAM) family enzyme [13,43]. The presence of a rSAM family enzyme in the BCG of *P. lentus* P8CEA5 encoding the mature bacteriocin supports the existence of 4 cysteine sulfur to α-carbon bridges in this bacteriocin. Given the significant identity differences (less than 60%) with other reported sactipeptides, this novel sactipeptide encoded by *P. lentus* P8CEA5 will be designated as lentucin S.

The presence of a peptide peak in the purified CFS of *P. lentus* P8CEA5 that resembles class IId thermophilin A from *Paenibacillus* spp., but with a 14 Da difference between the observed and predicted molecular mass (Figure 4), suggest methylation may have occurred on one of its amino acids, most likely associated with either lysine (K) or aspartic acid (D) [11,44]. Furthermore, the presence of two peptide peaks of 6150.86 and 6292.44 Da, may support the co-production by *P. lentus* P8CEA5 of two putative circular bacteriocins (PL1 and PL2) (Figure 4 and Figure S5). The class I circular or head-to-tail cyclized bacteriocins are RiPPs characterized by a peptide bond that links the N- and C-termini of the mature peptide. The BGC of circular bacteriocins includes genes encoding a bacteriocin precursor peptide, transporter protein(s), a SpoIIM (stage II sporulation protein M) membrane protein (previously known as DUF95), an immunity protein, and one or more unknown hydrophobic proteins (Figure S5). The lengths of the leader peptides can range from 2 to 48 amino acid residues [45,46].

Finally, the LC-MS/MS evaluation using targeted proteomics combined with massive peptide analysis of the RP-FPLC active fractions of the purified CFS of *P. lentus* P8CEA5, enabled the identification of a peptide fragment covering 48.4% of the sequence of the sactipeptide lentucin S, further supporting the production of this novel bacteriocin by this strain. Another identified peptide fragment covered 16.6% of the sequence of a Blp family class II bacteriocin thermophilin A LP. However, no peptide fragments corresponding to the putative circular bacteriocins PL1 and PL2 were observed.

The LC-MS/MS technique is a highly selective and sensitive method for detecting peptides in the low ng/mL to sub-ng/mL concentration range [47]. However, the relatively low-coverage percentage of peptide fragments identified in the Blp family class II bacteriocin LP, or their complete absence in the putative circular bacteriocins PL1 and PL2, are related to the very low presence of the circular bacteriocins in the purified CFS samples. Additionally, the reduced presence of lysine (K) and arginine (R) as trypsin cleavage sites in these bacteriocins significantly reduces the number of potentially identifiable target peptides. In summary, the results obtained indicate that *P. lentus* P8CEA5 produces a novel sactipeptide lentucin S, a class III lanthipeptide LP, a Blp family class IId bacteriocin thermophilin A LP, and two putative class I circular bacteriocins: PL1 and PL2.

In *S. saprophyticus* P1CEA4 the expression by using an IV-CFPS/SIML procedure of a putative class IIc (leaderless) bacteriocin, initially described an epidermicin LP, was achieved. This allowed for the determination of the antimicrobial activity of a 51-amino- acid bacteriocin with a calculated molecular mass of 6049.24 Da and a pI of 10.08 (Figure 2a). This bacteriocin is related but not identical (less than 70% identity), to other bacteriocins of the epidermicin NI01 and aureocin A53 family of bacteriocins produced by *Staphylococcus* spp. [48,49] (Figure S4), and has therefore been renamed saprophyticin S. While most bacteriocins are synthesized as inactive precursor peptides with an N-terminal leader peptide attached to a C-terminal propeptide, class IIc leaderless bacteriocins are characterized by the absence of an N-terminal leader peptide [50,51].

The *S. simulans* P8CEA7 strain was active against various indicator bacteria with the exception of *S. aureus*. This could be due to the the staphylococcal indicators sharing immunity or cross-resistance to the bacteriocins produced by *S. simulans* P8CEA7 [52]. Additionally, colony MALDI-TOF MS analysis of *S. simulans* P8CEA7 identified a class II lanthipeptide α LP with the hypothetical deletion of the last four amino acids, as well as the presence of a class II lanthipeptide β LP (Figure 3). In the absence of further information supporting the production of the bacteriocin lactococcin 972 LP encoded by *S. simulans* P8CEA7, it is possible that the class II lanthipeptide (α+β) LP contributes, at least in part, to the antimicrobial activity of this bacterial isolate.

Importantly, all the Gram-positive strains evaluated in this study showed no hemolytic and gelatinase activity. The prevalence of hemolytic and gelatinase activities in *L. salivarius* and *L. johnsonii*, is very low [53]. However, while hemolytic activity is likely minimal in *Paenibacillus* spp., gelatinase activity is often observed [54]. *S. saprophyticus* is non-hemolytic, which distinguishes it from other pathogenic staphylococci like *S. aureus* [55,56]. In contrast, *S. simulans* can exhibit both hemolytic and gelatinase activities, although the presence and extent of these activities can vary among different strains [57,58].

In this study, only lanthipeptides were identified after colony MALDI-TOF MS of the producer strains. Three different classes of lanthipeptides were detected: class I lanthipetide nisin S produced by *L. salivarius* P1CEA3, class I lanthipeptide paenicidin LP produced by *P. dendritiformis* P1CEA1, class II lanthipeptide (α+β) LP produced by *S. simulans* P8CEA7, and class III lanthipeptide LP produced by *P. lentus* P8CEA5. Therefore, this technique should be considered as effective for the rapid identification of class I, class II, and class III lanthipeptides encoded by different bacterial isolates [15,59].

The IV-CFPS protocol and the developed IV-CFPS/SIML procedure are considered effective methodologies for the rapid production and evaluation of antimicrobial activity in class IIb, IIc, and IId bacteriocins and class I circular bacteriocins, including those that are homologous, novel, or not yet described [15,60,61,62]. The IV-CFPS/SIML procedure for producing circular bacteriocins involves synthesizing the active mature bacteriocin through the association of inteins (I_C_ and I_N_), followed by splicing to yield the cyclic peptide product while liberating I_C_ and I_N_ as byproducts. This intein chemistry requires the first amino acid of the target peptide to preferably be a nucleophilic cysteine (C) or a serine (S) residue [61]. Consequently, the lack in this study of the synthesis of the putative circular peptides PL1 and PL2 by *P. lentus* P8CEA5, may be attributed to a lower tolerance of the inteins to amino acid near the splice junctions, and to significant peptide degradation. Further work will focus on improving this technology by evaluating more efficient and faster inteins and reducing peptide degradation [61].

## 4. Materials and Methods

### 4.1. Samples Collection, Isolation of Bacterial Strains and Growth Conditions

Samples were collected from the gastrointestinal tract (GIT) of pigs (Large White x Landrace) slaughtered in two slaughterhouses in the Community of Madrid (Spain). The first samples, collected at Getafe slaughterhouse (Getafe, Madrid, Spain), were from the content of the end of the GIT (rectum) of a batch of 100 pigs by collecting them with a stool vacuum cleaner and deposited into a tank from where the samples were collected. The second set of samples, collected at the Comaran S.L. slaughterhouse (Aranjuez, Madrid, Spain), were collected from the small intestine, cecum and colon of the GIT of nine pigs. Samples were collected in March 2021 from the slaughterhouse in Getafe and in July 2021 from the slaughterhouse in Aranjuez. The pigs were slaughtered and eviscerated following the regulatory practices established by Council Regulation (EC) No. 1099/2009 and Commission Implementing Regulation (EU) 2019/627 of the European Union (EU). Intestinal content samples were collected using sterile techniques, and all sampling equipment was sterilized prior to use. Samples were collected in 50 mL Falcon tubes, handled and transported hygienically at the laboratory and kept frozen at −80 °C.

A total of 0.1 g of each sample was homogenized with 1 mL of peptone water (Oxoid Ltd., Basingstoke, UK) and then serially diluted 1:10 in peptone water. Then 100 μL of each dilution was plated in MacConkey (Oxoid Ltd.) agar and Salmonella-Shigella (Oxoid Ltd.) agar for the isolation of Gram-negative bacteria, and in Brain Heart Infusion (BHI) (Oxoid Ltd.) agar, de Man, Rogosa and Sharpe (MRS) (Oxoid Ltd.) agar, and Baird-Parker (Oxoid Ltd.) agar for the isolation of Gram-positive bacteria. The plates were incubated at 37 °C overnight in aerobic conditions, except for those seeded on MRS agar, which were maintained in anaerobic conditions for 48 h. Selected isolates from the different plates were handpicked and inoculated into 96-well plates containing 250 μL of Luria Bertani (LB) (Sigma-Aldrich, Inc., St. Louis, MO, USA) broth for Gram-negative isolates, and BHI or MRS broth for Gram-positive isolates. These plates remained at 37 °C overnight in aerobiosis, except for those seeded in MRS which were maintained in anaerobiosis. Finally, glycerol was added to the wells at a final concentration of 30%, and the 96-well plates maintained frozen at −80 °C until further use.

### 4.2. Antimicrobial Activity Assays

Aliquots of the 96-well plates containing the collection of Gram-negative isolates were transferred to plates with LB broth using a 96-pin microplate replicator (Boekel Scientific, Feasterville-Trevose, PA, USA). These were incubated overnight at 37 °C in aerobic conditions. The cultures were then grown again in plates with M9 minimal broth (12.8 g/L Na_2_HPO_4_ x7H_2_O, 3 g/L KH_2_PO_4_, 0.5 g/L NaCl, 1 g/L NH_4_Cl, 2 mM MgSO_4_, 0.1 mM CaCl_2_, 0.4% [p/v] 0.5 mM thiamine, and 8 g/L D-glucose) at 37 °C in aerobiosis. When the cultures reached an OD_600_ of approximately 0.1, mitomycin C was added at a final concentration of 0.25 μg/mL per well, and the plates incubated again at 37 °C in aerobic conditions for 3 h. Then, 2–5 µL of the grown cultures were seeded, by using a 48-pin microplate replicator (Boekel Scientific, Feasterville-Trevose, PA, USA), into LB agar plates further maintained at 37 °C in aerobiosis. Colony growth was inhibited by using chloroform vapors for 30 min, followed by a 1 h standing period. The agar plates containing colonies were then overlaid with 5 mL of soft agar (0.8%, *w*/*v*) of the corresponding medium seeded with an overnight culture of the indicator bacteria (approximately 10^5^ CFU/mL). After another overnight incubation under appropriate conditions for each indicator strain (Table S1), the antimicrobial activity of the isolates was measured as the diameter of the inhibition zones in millimeters.

Similarly, aliquots from the 96-well plates containing Gram-positive bacterial isolates were transferred to plates with BHI or MRS agar using a 96-pin microplate replicator (Boekel Scientific), and incubated at 37 °C in aerobic and anaerobic conditions, respectively. Colony growth was again inhibited by using chloroform vapors for 30 min, followed by a 1 h-standing period. The agar plates were then overlayed with 5 mL of soft agar (0.8%, *w*/*v*) of the corresponding medium seeded with an overnight culture of the indicator microorganism (ca. 10^5^ CFU/mL). Following another overnight incubation of the plates at the appropriate conditions for each indicator strain (Table S1), the antimicrobial activity of the isolates was assessed by measuring the diameter of the halos of inhibition in millimeters. The bacterial indicators tested included Gram-negative bacteria (*Escherichia coli* and *Salmonella* spp.) and Gram-positive bacteria (*Staphylococcus aureus*, and *Streptococcus suis*) of porcine origin. Additionally, two indicator microorganisms, *E. coli* DH5α and *P. damnosus* CECT 4797, were included for their known sensitivity to a wide range of bacteriocins.

### 4.3. DNA Isolation and Purification, RAPD-PCR, and 16S rDNA Sequencing for Identification of the Most Active Bacterial Isolates

The Gram-negative and Gram-positive isolates with the highest antimicrobial activity and spectrum were grown overnight on their appropriate agar media (Table S1), DNA was isolated and purified from single colonies using an InstaGen Matrix (BioRad Laboratories, Inc., Hercules, CA, USA). The purified DNA from the selected isolates was then subjected to random amplification of polymorphic DNA by PCR (RAPD-PCR) using the low-specificity primer OPL5 (5′-ACGCAGGCAC-3′). The PCR was conducted in a total volume of 25 µL, comprising 12.5 µL of DreamTaq Green PCR Master Mix 2X (Thermo Fisher Scientific, Waltham, MA, USA), 0.5 µL of OPL5, 1 µL of template DNA, and molecular biology-grade water. The resulting PCR products were analyzed by agarose gel electrophoresis (2%, *w*/*v*) at 90 V/cm for 90 min, using HyperLadder 100 bp (Bioline, Meridian Bioscience, Inc., Cincinnati, OH, USA) as a molecular weight marker. The gels were stained with GelRed and visualized using a ChemiDoc Imaging System (BioRad Laboratories, Inc.).

Subsequently, the bacterial isolates showing a different pattern by RAPD-PCR, were taxonomically identified by amplification of their purified DNA with primers for amplification of a variable region of the 16S rDNA gene. PCR was performed in a total volume of 50 µL using the NZYTaq II 2X Master Mix (Nzytech, Lisboa, Portugal), 10 µM of primers rD1 (5′-TAAGGAGGTGATCCAGCC-3′) and fD1 (5′-AGAGTTTGATCCTGGCTCAG-3′) (Thermo Fisher Scientific) [63], 1 pg–100 ng DNA template, and molecular biology–grade water. The PCR products containing the HyperLadder 50 bp (Bioline) as a molecular weight marker were separated by agarose gel electrophoresis (1.5%, *w*/*v*) at 90 V/cm for 90 min, stained with GelRed and visualized using the ChemiDoc Imaging System (BioRad Laboratories, Inc.). PCR products were purified using the NucleoSpin^®^ Gel and PCR Clean-up kit (Macherey-Nagel GmbH & Co., Dueren, Germany), quantified with a NanoDrop (Thermo Fisher Scientific), and subjected to Sanger sequencing (Eurofins Genomics, Ebersberg, Germany). Taxonomy was inferred by searching the resulting 16S rDNA gene sequences for sequence similarity with the NCBI 16S rDNA database using the BLASTn tool (https://blast.ncbi.nlm.nih.gov/Blast.cgi, accessed on 3 November 2021).

### 4.4. Genomic DNA Isolation, Whole Genome Sequencing (WGS) and Assembly, Genome Annotation and Species Identification

Total genomic DNA was extracted from the 20 isolates of greatest interest, selected for their antimicrobial effects and spectrum of activity, as well as their taxonomic relevance, using the DNeasy Blood & Tissue Kit (Qiagen, Hilden, Germany). The purified DNA was quantified with a Qubit fluorometer (Invitrogen, Thermo Fisher Scientific), and its quality was confirmed by agarose gel electrophoresis (0.8%, *w*/*v*) stained with GelRed, using a 1 kb DNA Ladder (Sigma-Aldrich) as a molecular weight marker. The electrophoresis was conducted at 90 V/cm for 60 min, and visualization was performed using the ChemiDoc gel imaging system (BioRad Laboratories, Inc.). PCR products were purified using the NucleoSpin^®^ Gel and PCR Clean-up kit (Macherey-Nagel). Whole genome sequencing (WGS) of the purified genomic DNA was carried out at SeqCenter (Pittsburgh, PA, USA). Sample libraries were prepared using the Illumina DNA Prep kit, along with Integrated DNA Technologies (IDT) 10 bp unique dual index (UDI) indices. Sequencing was performed on an Illumina NextSeq 2000 (Illumina, San Diego, CA, USA), generating 2 × 151 bp reads. Demultiplexing, quality control, and adapter trimming were conducted using BCL Convert v3.9.3 (Illumina). The sequencing reads were processed and assembled using the Pathosystems Resource Integration Center (PATRIC) [64]. The resulting sequence reads were assembled into contigs with Unicycler v0.4.8 [65]. Assembly polishing was performed using Pilon (Oxford Nanopore Technologies, Oxford, UK). Coding sequences (CDS) were predicted and annotated using the Rapid Annotation Subsystem Technology (RAST) online server (http://rast.nmpdr.org/, accessed on 22 February 2022) [66]. Bacterial species identification was confirmed using KmerFinder v.3.0.2 (https://www.genomicepidemiology.org/services/, accessed on 22 February 2022), which utilizes a K-mer algorithm for species prediction [67].

### 4.5. Bioinformatic Screening of Biosynthetic Gene Clusters Encoding Bacteriocins

From the collected WGS data of the most active isolates, an extensive bioinformatics analysis was performed to identify biosynthetic gene clusters (BGC) involved in the production of non-modified bacteriocins and ribosomally synthesized and post-translationally modified peptides (RiPPs). The assembled genomes were analyzed using default settings in the online tools BAGEL v4.0 (http://bagel4.molgenrug.nl/, accessed on 3 March 2022) [68] and the Antibiotics and Secondary Metabolite Analysis Shell (antiSMASH) (https://antismash.secondarymetabolites.org/, accessed on 3 March 2022) [69]. To confirm the identity of the putative bacteriocins identified, SnapGene v7.0.3 software (GSL Biotech, San Diego, CA, USA), BLASTp (NCBI) (https://blast.ncbi.nlm.nih.gov/Blast.cgi/, accessed on 3 March 2022) [70], and the UniProt database (https://www.uniprot.org/, accessed on 10 March 2022) [71] were utilized. Isolates encoding similar or identical bacteriocins underwent analysis using Mauve v2.4.0 for multiple genome alignment [72] to assess genome similarities.

### 4.6. Hemolytic and Gelatinase Activities

The hemolytic and gelatinase activities of the selected strains were assessed in vitro, as previously described [73]. Hemolysin production was evaluated by growing the strains in their respective broth media under appropriate conditions (Table S1), and then streaked on horse blood agar plates (BioMérieux, Marcy-l’Étoile, France), which were incubated for 24 h. The presence of clear zones of hydrolysis around the colonies indicated β-hemolysis. Gelatinase production was evaluated by growing the strains in their appropriate broth media and conditions (Table S1), followed by streaking onto Todd–Hewitt (Oxoid Ltd.) agar plates (1.5%, *w*/*v*) supplemented with 30 g of gelatin per liter. The plates were incubated for 16 h, then stored at 4 °C for 5 h. Turbid zones around the colonies, indicative of protein hydrolisis, were examined. In both assays, *E. faecalis* P4 and *E. faecalis* SDP10 served as positive controls for hemolysin production and gelatinase activity, while *L. lactis* subsp. *lactis* BB24 was used as the negative control.

### 4.7. In Vitro Cell-Free Protein Synthesis (IV-CFPS) of Mature Bacteriocins and Evaluation of Their Antimicrobial Activity

Total genomic DNA from selected strains was used as a template for PCR amplification of genes encoding putative mature class II bacteriocins, utilizing specific primers (Table S2). Forward and reverse primers were designed according to the recommendations of the PURExpress^®^ In Vitro Protein Synthesis Kit (New England Biolabs, Ipswich, MA, USA), incorporating the T7 promoter and transcription terminator regions, as previously described [15,60]. The oligonucleotide primers were obtained from Thermo Fisher Scientific. PCR amplifications were conducted with Phusion Hot Start II High-Fidelity DNA Polymerase (Thermo Fisher Scientific) in 50 µL reaction mixtures containing 5–100 ng of purified DNA and molecular biology-grade water. The amplification conditions were as follows: initial denaturation at 98 °C for 30 s; 35 cycles of denaturation (98 °C for 10 s), annealing (60 °C for 10 s), and elongation (72 °C for 15–30 s per kb); followed by a final elongation at 72 °C for 5 min in a thermal cycler (Eppendorf, Hamburg, Germany). PCR amplicons were visualized by agarose gel electrophoresis using a ChemiDoc Imaging System (BioRad Laboratories, Inc.). The PCR-derived products were purified using the NucleoSpin^®^ Gel and PCR Clean-up kit (Macherey-Nagel) and quantified with a Qubit fluorometer (Invitrogen, Thermo Fisher Scientific).

Purified PCR-derived amplicons were then used as templates for the in vitro cell-free protein synthesis (IV-CFPS) of the bacteriocins of interest, at a final concentration of 10 ng/µL in 25 µL reactions using the PURExpress In Vitro Protein Synthesis Kit (New England Biolabs). The reactions were maintained at 37 °C for 2 h and then placed on ice to stop the reaction. Carrier plasmids with synthetic genes encoding both native and putative mature class II bacteriocins, along with the T7 promoter and transcription terminator regions, were also designed and employed as templates for the IV-CFPS of the corresponding bacteriocins, maintaining the same conditions (10 ng/µL in 25 µL reactions, 37 °C for 2 h, then placed on ice). The antimicrobial activity of the IV-CFPS reactions was assessed using the spot-on-agar test (SOAT), as previously described [60]. This involved depositing 5 μL samples onto the surface of Petri plates overlaid with a soft agar (0.8%, *w*/*v*) culture of the indicator microorganism (approximately 10^5^ CFU/mL). The plates were then incubated under appropriate conditions for growth of the indicator strain. Chemically synthesized class II bacteriocin bactofencin A, provided by Syngulon (Seraing, Belgium) at a concentration of 1 mg/mL and a purity of 95–99% was also evaluated for its antimicrobial activity using the SOAT.

### 4.8. In Vitro Cell-Free Protein Synthesis (IV-CFPS) Coupled to a Split-Intein-Mediated Ligation (SIML) Procedure for Synthesis, Production and Determination of the Antimicrobial Activity of Putative Circular Bacteriocins

Putative circular bacteriocins PL1 and PL2, encoded in the genome of *P. lentus* P8CEA5, were synthesized in vitro using an IV-CFPS protocol coupled to a split-intein-mediated-ligation (IV-CFPS/SIML) procedure for ligation of circular bacteriocins [61,62]. Briefly, two synthetic gene constructs featuring the C- and N-terminal fragments the C- and N-terminal fragments from the *Nostoc puntiforme* (Npu) DnaE split-intein, flanking the gene coding the putative circular bacteriocins PL1 and PL2, were designed. For both bacteriocins, serine at position 34 (S34) was selected as the first amino acid residue in the linear conformation. These synthetic genes were placed under the control of a pUC-derived protein expression vector containing a T7 promoter, a start codon (ATG), a stop codon (TAA), and a T7 transcription terminator. The resulting gene constructs, pCirc-PL1 and pCirc-PL2, were obtained from GeneArt (Life Technologies/Thermo Fisher Scientific, Waltham, MA, USA). Both plasmids were used as templates for the IV-CFPS/SIML reaction at a final concentration of 10 ng/µL in 25 µL reactions, maintained at 37 °C for 2 h, and then placed on ice to stop the reaction. The antimicrobial activity of the IV-CFPS/SIML-derived reactions was evaluated using the SOAT against the indicator strains *P. damnosus* CECT 4797, *L. seeligeri* CECT 917, *B. cereus* ICM17/00252, *B. pumilus* PE12, *B. toyonensis* NM11 and *P. dendritiformis* P1CEA1.

### 4.9. Colony Matrix-Assisted Laser Desorption/Ionisation-Time of Flight Mass Spectrometry (MALDI-TOF MS) Analysis

Bacterial strains predicted to encode class I, class II, and class III lanthipeptides were analyzed using colony MALDI-TOF MS analysis, as previously described [15]. Briefly, single colonies of the strains, grown under appropriate conditions, were harvested and mixed with 50 μL of 100% isopropanol containing 0.1% (*v*/*v*) trifluoroacetic acid (TFA). The mixture was vortexed three times and centrifuged at 13,000 rpm for 30 s. Subsequently, 1 μL of the supernatant was combined with 1 μL of either a sinapic acid matrix or α-cyano-4-hydroxycinnamic acid (Sigma-Aldrich), both dissolved in 30% acetonitrile and 0.3% TFA. This mixture was then directly applied to the MS target plate and dried using a stream of warm air. MALDI-TOF MS analysis of the samples was performed on an Ultraflex workstation (Bruker Daltonics, Billerica, MA, USA) equipped with a 337 nm nitrogen laser at the Unidad de Espectrometría de Masas (CAI Técnicas Químicas, UCM, Madrid, Spain). The mass spectrometer was calibrated using Protein Calibration Standard I (4000–20,000 *m*/*z*) from Bruker Daltonics. Sample analysis and method parameter control were conducted using FlexControl Software v.2.4 (Bruker Daltonics).

### 4.10. Growth Conditions and Antimicrobial Activity of Paenibacillus lentus P8CEA5

To optimize the growth and synthesis of bacteriocins by the multi-encoding bacteriocin producer *P. lentus* P8CEA5, several growth conditions were evaluated. These included growth in TSB and BHI broth, aerobic and anaerobic environments, stirred versus static culture, and temperatures of 32 °C and 37 °C. Cultures were incubated overnight, and the CFS was obtained by centrifuging the cultures at 12,000× *g* for 10 min at 4 °C, followed by filtration through 0.22 μm pore-size syringe filters. The antimicrobial activity of the CFS was assessed using the SOAT, in which 5 μL of each CFS was added to appropriate agar plates previously overlaid with 5 mL of soft agar (0.8%, *w*/*v*) seeded with an overnight culture of the selected indicator strain (approximately 10^5^ CFU/mL). After overnight incubation at 37 °C, the diameters of the inhibition halos were measured in millimeters.

### 4.11. Purification of the Bacteriocins Produced by P. lentus P8CEA5, MALDI-TOF MS Analysis, and LC-MS/MS Evaluation by Targeted Proteomics Combined with Massive Peptide Analysis of the Trypsinized Purified Fractions

Bacteriocins were purified from 1 L cultures of *P. lentus* P8CEA5, grown at 37 °C for 48 h in TSB and centrifuged at 12,000× *g* for 10 min at 4 °C to obtain the corresponding CFS. Next, the CFS underwent ammonium sulfate precipitation, desalting by gel filtration, and hydrophobic-interaction chromatography, followed by two rounds of reverse-phase chromatography using an ÄKTA Purifier fast protein liquid chromatography (RP-FPLC) system, as previously described [15,62]. In the second round of RP-FPLC the sample was loaded onto a SOURCE 5RPC ST 4.6/150 column (Cytiva, Marlborough, MA, USA), and the retained compounds eluted with a gradient of 0% to 100% isopropanol (Thermo Fisher Scientific) with 0.1% (*v*/*v*) trifluoroacetic acid (TFA) (Panreac, Madrid, Spain). The eluted fractions were monitored at 254 nm (A_254_), and their antimicrobial activity was evaluated using the SOAT against the indicator microorganisms *P. damnosus* CECT 4749, *L. seeligeri* CECT 917, and *B. cereus* ICM17/00252. The CFS fractions exhibiting the highest antimicrobial activity were analyzed for molecular mass using MALDI-TOF MS at the Unidad de Espectrometría de Masas (CAI Técnicas Químicas, UCM, Madrid, Spain), as detailed in Section 4.9 of the manuscript.

Additionally, the purified RP-FPLC fractions from the CFS of *P. lentus* P8CEA5 were subjected to LC-MS/MS analysis at the Unidad de Proteómica (CAI Técnicas Biológicas, UCM, Madrid, Spain). In this process, the peptides and proteins in the samples were digested with trypsin using S-Trap™ micro columns (ProtiFI, Fairport, NY, USA) according to the manufacturer’s instructions (Roche Molecular Biochemicals, NJ, USA). Approximately 1 µg of the resulting peptides was analyzed using liquid nano-chromatography on a Vanquish Neo (Thermo Fisher Scientific) coupled with a Q-Exactive HF high-resolution mass spectrometer (Thermo Fisher Scientific). The peptides were eluted over a 30-min gradient from 2% to 35% buffer B (0.1% formic acid in 2% acetonitrile) in buffer A (0.1% formic acid in dH2O) at a constant low flow rate of 250 nL/min. Data acquisition on the Q-Exactive HF was conducted using a targeted proteomics approach to identify the peptides of interest in the samples. Peptide identifications from the MS/MS data were carried out using Proteome Discoverer 2.4 software (Thermo Fisher Scientific) with the MASCOT v2.6 or Sequence HT search engines, along with Peaks Studio v10.5 software (Bioinformatic Solutions Inc., Waterloo, ON, Canada), which includes additional tools such as de novo sequencing to maximize peptide and protein identification.

## 5. Conclusions

Commercial bacterial replicators streamline the assessment of the antimicrobial activity of Gram-negative and Gram-positive isolates sourced from meat-producing pigs. WGS of the most active isolates enabled identification of BGC and their encoded bacteriocins. Most bacterial strains held BGC in their genomes, except *L. reuteri* P1CEA2 and *L. reuteri* P8SIA3. Most Gram-negative strains encoded previously described colicins and microcins. *E. coli* PG14 and *E. coli* PG15 encoding identical bacteriocins, showed a distinct conformation of adjacent open reading frames (ORF) in their BGC. The IV-CFPS protocol and IV-CFPS/SIML procedure allowed the evaluation of described and novel class IIb, class IIc, class IId, and class I circular bacteriocins in the bacteriocin-producing *L. salivarius* P1CEA3, *L. salivarius* PG21, *S. saprophyticus* P1CEA4 and *P. lentus* P8CEA5 isolates. Colony MALDI-TOF MS confirmed the production of class I, II, and III lanthipeptides by *L. salivarius* P1CEA3, *P. dendritiformis* P1CEA1, *P. lentus* P8CEA5, and *S. simulans* P8CEA7. MALDI-TOF MS, and LC-MS/MS revealed that *P. lentus* P8CEA5 produces a novel class I sactipeptide lentucin S, the class III lanthipetide LP, the class IId Blp family bacteriocin LP, and two novel class I circular bacteriocins: PL1 and PL2. The identified bacteriocins are promising candidates for food preservation and therapeutic applications, while the bacteriocin-producing strains offer promising prospects for evaluation as probiotics in animal production.

## Figures and Tables

**Table 1:** Antimicrobial effect ^a^ and spectrum of activity of selected Gram-negative isolates, against different indicator strains.

Producer Strains	Indicator Strains									
	*E. coli*						*S.* Choleraesuis			*S. paratyphi*
	DH5α	ZTA16/ 01940	ZTA16/ 01878	ZTA16/ 01937	ZTA16/ 01268	ZTA16/ 02317	ZTA19/ 01344	ZTA19/ 01349	ZTA19/ 01351	CECT 554
***P. alcaligenes* PG7**	+	++	+	-	-	-	-	-	-	-
***E. coli* PG9**	++	++	++	+	++	++	-	-		-
***E. coli* PG14**	+++	+	+	+	+	-	-	-	-	-
***E. coli* PG15**	-	-	-	-	-	-	++	+	+	-
***E. coli* PG18**	+	-	-	+	-	-	-	-		-
***E. coli* P8CEA3**	++	++	+	++	+	-	-	-	-	-
***E. coli* P8COA2**	+++	+++	+++	+	+++	+++	-	-	-	++

^a^ Antimicrobial activity calculated as the diameter of halos of inhibition, -: no inhibition, +: <10 mm, ++: 10–20 mm, +++: >20 mm.

**Table 2:** Antimicrobial effect ^a^ and spectrum of activity of selected Gram-positive isolates, against different indicator strains.

Producer Strains	Indicator Strains					
	*P. damnosus* CECT 4797	*S. aureus* ZTA11/00117ST	*S. aureus* ZTA11/00310ST	*L. seeligeri* CECT 917	*S. suis * C2969/03	*S. suis* CECT 958
***L. reuteri* P1CEA2**	+	-	-	-	-	-
***L. reuteri* P8SIA3**	++	+++	+++	+++	+++	+++
***L. salivarius* P1CEA3**	+++	+	+	++	++	++
***L. salivarius* PG21**	+++	+++	+++	+++	+++	+++
***L. johnsonii* P8CEA12**	++	++	+	++	+	+
***L. johnsonii* P8COA6**	+++	++	++	+++	++	++
***L. johnsonii* P8COA7**	+	++	++	+++	+	+
***P. dendritiformis* P1CEA1**	+++	++	++	++	++	++
***P. lentus* P8CEA4**	-	+	+	++++	+	++
***P. lentus* P8CEA5**	-	+	+	++++	+	++
***P. lentus* P8SIA1**	-	+	+	+++	+	+
***S. saprophyticus* P1CEA4**	+++	+	+	++	++	++
***S. simulans* P8CEA7**	+++	-	-	+++	+++	+++

^a^ Antimicrobial activity as the diameter of halos of inhibition, -: no inhibition, +: <10 mm, ++: 10–20 mm, +++: >20 mm.

**Table 3:** Bacteriocins identified, along with the amino acid sequences of the novel identified hypothetical bacteriocins encoded by the most active Gram-negative strains identified in this study. X indicates the presence of the suggested bacteriocin gene.

Strain	S-Type Pyocin	BLP	Colicin							Microcin			
			E6	Ia	Ib	E1	E2	S4	10	H47	M	V	B17
*P. alcaligenes* PG7	X												
*E. coli* PG9		X	X	X		X							
*E. coli* PG14				X						X	X	X	
*E. coli* PG15				X						X	X	X	
*E. coli* PG18					X								
*E. coli* P8CEA3						X						X	X
*E. coli* P8COA2		X			X		X	X	X				
	**Amino Acid Sequence**												
**S-type pyocin**	MTLLRRIDMSGYVANNRDVRSAPEIPVYNGAFDQQPRRQTDRPSPLMPEPLPVPPTQCVFAKPNSLPLGSLDYPSVVPAELASAYGQTAILATTDVPAAGGGLLLARASGQLVGGGTWAIQSAAGAGGTAAGSGATGAAGSGILATAATTAIGFVALLWPSPMGSSDLYPKSELEVLSTAKTRLRFHVEHDWVNGSIRTYGFHTSSRSGFDSVPVVAARAQGEQAVVDLGDGVTILWTPQVDPAVGAPPPPEDIQGLTETVWIYPVSQNAAQALENPIYPSDYKDFIITFPDHPGVQPVYVVLSTQLEKNKVRGREFEDEVYGDYSSTRSETGREVTVKTKSGTRTRIDMVGREPDGTISCVECKSSDTAPLTPNQKVAFPEIEESGAVVVGKGKPGFPGGTEIPPTKVDVVRPN												
**BLP**	MGYGLLDIANQSRREALQGISDADRRREEIEAANKQMAAQQKAQNKQNIGTGIGTGAAIGASVGGPVGAVAGAVIGGIAGSLF												

**Table 4:** Identified bacteriocins, their names, and amino acid sequence of the bacteriocins encoded by the most active Gram-positive strains identified in this study.

Identification	Strain	Bacteriocin ^a^	Amino Acid Sequence ^b^
*Limosilactobacillus reuteri*	P1CEA2	-	-
*Limosilactobacillus reuteri*	P8SIA3	-	-
*Ligilactobacillus salivarius*	P1CEA3	Salivaricin B	MNNNFVQVDKKELAHIIGGRNSYDYIDSGQFGYDIGCTIANTKFFKRLRHSNQNICS
		Abp118α	MIIMMKEFTVLTECELAKVDGGKRGPNCVGNFLGGLFAGAAAGVPLGPAGIVGGANLGMVGGALTCL
		Abp118β	MKNLDKRFTIMTEDNLASVNGGKNGYGGSGNRWVHCGAGIVGGALIGAIGGPWSAVAGGISGGFTSCR
		Nisin S (class I)	MSVNDFKLDLVKVSKESTNSNYSVKITSYSLCTPGCKTGALMGCTMKTASCGCHVHISK
*Ligilactobacillus salivarius*	PG21	Bactofencin A	MFFNFMKKVDVKKNFGYKEVSRKDLAKVNGGKRKKHRCRVYNNGMPTGMYRWC
		Salivaricin Tα LP	MIIMMKEFTVLTECELAKVDGGYTPKNCAMAVGGGMLSGAIRGGMSGTVFGVGTGNLTGAFAGAHIGLVAGGLACIGGYLGSH
		Salivaricin Tβ LP	MSYEKLNNEELSKILGGNGINWGAVVGSCASGAVIGAAFGNPLTGYVANSAFSFSWQAFKNRPHPKKIA
		Gassericin T/LactacinF lafA LP	MIIMMDKEFTVLTECELAKVDGGKGSKGSSYVAGFASAAIADTGLGGAICGVPCAMIGAHYAPIGWTIVTGATGGF
		Plantaricin NC8α LP	MKVYNEENLAEIIGGRSIEGKIWYGYGYQLGMTARWNLRHPYFQLPYH
		Plantaricin NC8β LP	MNKKLNSIDEKDLVKIVGGGSPWSNLIVQGAVAVFKSGYRHRNDIKAGFSAGFYGK
		Plantaricin Sα LP	MITMNNLQKFEIISDTTLSHVNGGYNRLAGRIGHYTGKAALWGIAVAGLFLI
		Plantaricin Sβ LP	MDNCNNFTSLNNTELQGIIGGKHGLGYHIVDAVVSFGEGFLNAI
*Lactobacillus johnsonii*	P8CEA12 P8COA7 P8COA6	Helveticin J LP	MIGRETQICLVNKLENIHHVVVQASAIDGSNVFALQLLHKQSDVVVYQTPNDSETVTFDEDHPILYLKGPNSAGTAGGHTQTWIQSGENNKWFVGTKPKRQGNTYWTTQIARVTVPGYQTQVFANNTDLPRLSYLNRAGAGYGDGGTVYPGKDLVRVEATVSPNGHYFLIASIDINHTGYFALYDLNEVNNKLDAAEEKAEDINIETLTCLGAFKVPHFNDQKIISIQGYGIDDNKDIYISSQPSPHTTFLGFPRQGKPREIVKIPWGMVDPDKWSVVNLDNSLKLDALDFCTEFEGIQVTSDCLYLTVAYHQRNSDLTTLMNRIYQVEKF
*Paenibacillus dendritiformis*	P1CEA1	Proteusin peptide LP	MSIDQMHMNQIVQRAWEDATFKEKLLADPKSAIKELLGISIPEHIQLTTVEEKLNQYVLILPPNPSEVVKDTPVAKRDMWY
		Lasso peptide paeninodin	MKKQYSKPSLEVLDVHQTMAGPGTSTPDAFQPDPDEDVHYDS
		Class I lanthipeptide paenicidin LP	MENNLFDLDIQVKKSSDNIEPQVLSVWRCSGGCSDDGRTSATCVATAGKSCFTNIGSLC
		Heterocycloanthracin/sonorensin LP	MSDFQKDLQNLNVGKFSSTGMTPASGQNQSGPGDPRLCVGICFFCIGFCGGFCSCGNCFRCSNCFRCSNCFNCANCSNCARCGSCARCR
*Paenibacillus lentus*	P8CEA4 P8CEA5 P8SIA1	Proteusin peptide LP	MTTSGALLQTQIIQKAWQDPSFKAKLLADPKAAIQEVLGVTIPDHIKVKTIEENSDEFYLVLPPNPSEVVKSDIKPNAVWGN
		Sactipeptide thuricin 17 LP (lentucin S)	MVTPSVEPNGITCWGCLACAACTVTALTLVSALSGLNTID
		Circular bacteriocin PL1	MRAQILKKDTKLFISMSLVVVLSSMLLFTFKFAAVNLGLSASTATLLYTALNAIAWGATAAGIVASFGLGAIAAQAIWSYVKKHTLKQFLQY
		Circular bacteriocin PL2	MVLKKDSKAIFFVSLSILLSTLLLMNFHNAAVTIGISEGLATNIYFALQWVSWGATAAAIVASFGLGAIVAQTIWAAVKKQALKEFVKW
		Class III lanthipeptide LP	MNDVLALQQLAAAPEEQELFPITITWTVTTTVATWSTASNHC
		Thermophilin A LP	MDSVMQLNGFSELSLNEMMSIDGGADFSWKDLGKSVVGGAASGAIGGGAAGAMAGGVGAGPGALVGGAAGAVGGAAAYLLTYWW
*Staphylococcus saprophyticus*	P1CEA4	Epidermicin LP (saprophyticin S)	MGAFLKFVGWLATKGKKYVKIAWDHKGTIMKWLNAGQTFTWVYEQIKKLWT
*Staphylococcus simulans*	P8CEA7	Lactococcin 972 LP	MKKYVARTIIIATLLLGMGTTTIANAYEWAEGGKWSHGIGSTYVWSYYTHNSYGHDSTAIGKYRSDSGYTTAGKQARASAKKAWWGNQAYYRVY
		Class II lanthipeptide α LP	MTKKDLLSSAKKDYLEKVDISDDKLEDVQGGKCPWYNVSCQLGNDGKICTFSHECTGGCNTSA
		Class II lanthipeptide β LP	MSLFKKDIKRNVNAENNLKKVSNVEDKQGGTTTVPCAVAASVALCPTLVCSNKCGDRG

^a^ LP, stands for like-peptide, indicating that the bacteriocin is similar but not identical. ^b^ In the amino acid sequence of the bacteriocins, the signal peptide sequence is underlined, while the mature sequence is in plain letters.

## Data Availability

The whole genome assembly of the selected Gram-negative and Gram-positive bacteriocinogenic isolates is deposited in NCBI under the Bioproject accession number PRJNA1160269.

## Supplementary Materials

The following supporting information can be downloaded at: https://www.mdpi.com/article/10.3390/ijms252212210/s1.
