# Supplementary material for: Isolation, Genomics-Based and Biochemical Characterization of Bacteriocinogenic Bacteria and Their Bacteriocins, Sourced from the Gastrointestinal Tract of Meat-Producing Pigs
Source: Int J Mol Sci. 2024 Nov 14;25(22):12210. doi: 10.3390/ijms252212210 (PMC11594732; doi:10.3390/ijms252212210)
Supplement: Supplementary file 1 [file ijms-25-12210-s001.zip › ijms-3297450-supplementary.pdf]

**Table S1.** Origin, growth medium and incubation conditions for the producer and indicator strains used in this study.

| Strain | Origin[a] | Growth medium | Incubation conditions | Use in this study |
|---|---|---|---|---|
| **Gram negative bacteria** | | | | |
| *Pseudomonas alcaligenes* PG7 | DNBTA | LB/M9 | 37 ºC/ Agitation | Producer |
| *Escherichia coli* PG9 | DNBTA | LB/M9 | 37 ºC/ Agitation | Producer |
| *Escherichia coli* PG14 | DNBTA | LB/M9 | 37 ºC/ Agitation | Producer |
| *Escherichia coli* PG15 | DNBTA | LB/M9 | 37 ºC/ Agitation | Producer |
| *Escherichia coli* PG18 | DNBTA | LB/M9 | 37 ºC/ Agitation | Producer |
| *Escherichia coli* P8CEA3 | DNBTA | LB/M9 | 37 ºC/ Agitation | Producer |
| *Escherichia coli* P8COA2 | DNBTA | LB/M9 | 37 ºC/ Agitation | Producer |
| *Escherichia coli* DH5α | Thermofisher | LB | 37 ºC/ Agitation | Indicator |
| *Escherichia coli* ZTA16/01940 | VISAVET | LB | 37 ºC/ Agitation | Indicator+PO |
| *Escherichia coli* ZTA16/01878 | VISAVET | LB | 37 ºC/ Agitation | Indicator+PO |
| *Escherichia coli* ZTA16/01937 | VISAVET | LB | 37 ºC/ Agitation | Indicator+PO |
| *Escherichia coli* ZTA16/01268 | VISAVET | LB | 37 ºC/ Agitation | Indicator+PO |
| *Escherichia coli* ZTA16/02317 | VISAVET | LB | 37 ºC/ Agitation | Indicator+PO |
| *Salmonella paratyphi* CECT 554 | CECT | BHI | 37 ºC/ Aerobiosis | Indicator |
| *Salmonella* Choleraesuis ZTA19/01344 | VISAVET | BHI | 37 ºC/ Aerobiosis | Indicator+PO |
| *Salmonella* Choleraesuis ZTA19/01349 | VISAVET | BHI | 37 ºC/ Aerobiosis | Indicator+PO |
| *Salmonella* Choleraesuis ZTA19/01351 | VISAVET | BHI | 37 ºC/ Aerobiosis | Indicator+PO |
| *Pseudomonas fluorescens* B52 | DNBTA | BHI | 37 ºC/ Aerobiosis | Indicator |
| *Pseudomonas putida* 3 | DNBTA | BHI | 37 ºC/ Aerobiosis | Indicator |
| **Gram positive bacteria** | | | | |
| *Limosilactobacillus reuteri* P1CEA2 | DNBTA | MRS | 37 ºC/ Anaerobiosis | Producer |
| *Limosilactobacillus reuteri* P8SIA3 | DNBTA | MRS | 37 ºC/ Anaerobiosis | Producer |
| *Ligilactobacillus salivarius* P1CEA3 | DNBTA | TSB/BHI/MRS | 37 ºC/ Anaerobiosis | Producer |
| *Ligilactobacillus salivarius* PG21 | DNBTA | MRS | 37 ºC/ Aerobiosis | Producer |
| *Lactobacillus johnsonii P8CEA12* | DNBTA | MRS | 37 ºC/ Aerobiosis | Producer |
| *Lactobacillus johnsonii* P8COA6 | DNBTA | MRS | 37 ºC/ Aerobiosis | Producer |
| *Lactobacillus johnsonii* P8COA7 | DNBTA | MRS | 37 ºC/ Aerobiosis | Producer |
| *Paenibacillus dendritiformis* P1CEA1 | DNBTA | TSB/BHI | 37 ºC/ Aerobiosis | Producer/ indicator+PO |
| *Paenibacillus lentus* P8CEA4 | DNBTA | TSB/BHI | 37 ºC/ Aerobiosis | Producer |
| *Paenibacillus lentus* P8CEA5 | DNBTA | TSB/BHI | 37 ºC/ Aerobiosis | Producer |
| *Paenibacillus lentus* P8SIA1 | DNBTA | TSB/BHI | 37 ºC/ Aerobiosis | Producer |
| *Staphylococcus saprophyticus* P1CEA4 | DNBTA | TSB/BHI | 37 ºC/ Aerobiosis | Producer |
| *Staphylococcus simulans* P8CEA7 | DNBTA | TSB/BHI | 37 ºC/ Aerobiosis | Producer |
| *Lactococcus lactis* subsp. *lactis* BB24 | DNBTA | MRS | 32 ºC/ Aerobiosis | Control |
| *Pediococcus damnosus* CECT 4797 | CECT | MRS | 32 ºC/ Aerobiosis | Indicator |
| *Enterococcus faecalis* P4 | DNBTA | MRS | 37 ºC/ Aerobiosis | Control |
| *Enterococcus faecalis* SDP10 | DNBTA | MRS | 37 ºC/ Aerobiosis | Control |
| *Listeria seeligeri* CECT 917 | CECT | BHI | 37 ºC/ Aerobiosis | Indicator |
| *Listeria monocytogenes* CECT 4032 | CECT | BHI | 37 ºC/ Aerobiosis | Indicator |
| *Staphylococcus aureus* ZTA11/00117ST | VISAVET | BHI | 37 ºC/ Aerobiosis | Indicator+PO |
| *Staphylococcus aureus* ZTA11/00310ST | VISAVET | BHI | 37 ºC/ Aerobiosis | Indicator+PO |
| *Streptococcus suis* CECT 958 | CECT | BHI | 37 ºC/ Aerobiosis | Indicator+PO |
| *Streptococcus suis* C2969/03 | VISAVET | BHI | 37 ºC/ Aerobiosis | Indicator+PO |
| *Bacillus cereus* ICM17/00252 | VISAVET | BHI | 37 ºC/ Aerobiosis | Indicator+PO |
| *Bacillus pumilus* PE12 | DNBTA | BHI | 37 ºC/ Aerobiosis | Indicator |

| | | | | |
|---|---|---|---|---|
| *Bacillus toyonensis* NM11 | DNBTA | BHI | 37 ºC/ Aerobiosis | Indicator |

[a] DNBTA: Departamento de Nutrición, Bromatología y Tecnología de los Alimentos, Facultad de Veterinaria, Universidad Complutense de Madrid (UCM), Madrid, (Spain). VISAVET: Centro de Vigilancia Sanitaria Veterinaria, Universidad Complutense de Madrid (UCM), Madrid, (Spain). CECT: Colección Española de Cultivos Tipo, Valencia, (Spain). PO, refer to indicator strains of porcine origin.

**Table S2**. Primers for amplification by PCR-mediated reactions of identified mature bacteriocins encoded by selected Gram-negative and Gram-positive strains, isolated in this study.

| Primers | Primers nucleotide sequence (5´- 3´) [a] | Amplification fragments | PCR products [b] | Strain |
|---|---|---|---|---|
| pyoPG7-F1 | **GCGAATTAATACGACTCACTATAGGGCTTAAGTATAAGGAGGAAAAAAT**ATGACTCTGCTTCGAAGGATCGATATG | S-type pyocin | S-type pyocin | *P. alcaligenes* PG7 |
| pyoPG7-F2 | **GCGAATTAATACGACTCACTATAGGGCTTAAGTATAAGGAGGAAAAAAT**ATGAGCGGCTACGTAGCGAACAACCGG | S-type pyocin | | |
| pyoPG7-R | AAACCCCTCCGTTTAGAGAGGGGTTATGCTAGTTAGTTCGGTCGTACAACGTCAACTTT | S-type pyocin | | |
| abp118α-F | **GCGAATTAATACGACTCACTATAGGGCTTAAGTATAAGGAGGAAAAAAATATG**AAACGTGGTCCTAACTGTGTAGGTAAC | abp118α | bacteriocin abp118α | *L. salivarius* P1CEA3 |
| abp118α-R | AAACCCCTCCGTTTAGAGAGGGGTTATGCTAGTTATTATAAACAAGTAAGTGCTCCGCC | | | |
| abp118β-F | **GCGAATTAATACGACTCACTATAGGGCTTAAGTATAAGGAGGAAAAAAATATG**AAAAATGGTTATGGTGGTAGTGGAAAT | abp118β | bacteriocin abp118β | |
| abp118β-R | AAACCCCTCCGTTTAGAGAGGGGTTATGCTAGTTAAAAAATGGTTATGGTGGTAGTGGA | | | |
| salB-F | **GCGAATTAATACGACTCACTATAGGGCTTAAGTATAAGGAGGAAAAAAATATG**AGAAATTCTTATGATTATATAGATAGC | salB | salivaricin B | |
| salB-R | AAACCCCTCCGTTTAGAGAGGGGTTATGCTAGTTAACTACAAATATTTTGATTTGAGTG | | | |
| salTa-F | **GCGAATTAATACGACTCACTATAGGGCTTAAGTATAAGGAGGAAAAAAT**ATGTATACGCCTAAAAACTGTGCAATGGCA | salTα | salivaricin Tα LP | *L. salivarius* PG21 |
| salTa-R | AAACCCCTCCGTTTAGAGAGGGGTTATGCTAGTTAATGACTACCTAAATATCCTCCGAT | | | |
| salTb-F | **GCGAATTAATACGACTCACTATAGGGCTTAAGTATAAGGAGGAAAAAAT**ATGAATGGCATTAATTGGGGAGCAGTCGTA | salTβ | salivaricin Tβ LP | |
| salTb-R | AAACCCCTCCGTTTAGAGAGGGGTTATGCTAGTTACGCTATTTTTTTAGGATGAGGTCT | | | |
| plantNC8a-F | **GCGAATTAATACGACTCACTATAGGGCTTAAGTATAAGGAGGAAAAAAT**ATGAGAAGTATAGAAGGTAAGATATGGTAT | plantNC8α | plantaricin NC8α LP | |
| plantNC8a-R | AAACCCCTCCGTTTAGAGAGGGGTTATGCTAGTTAATGATAAGGTAATTGAAAATACGG | | | |
| plantNC8b-F | **GCGAATTAATACGACTCACTATAGGGCTTAAGTATAAGGAGGAAAAAAT**ATGTCTCCATGGTCAAACCTCATTGTTCAA | plantNC8β | plantaricin NC8β LP | |
| plantNC8b-R | AAACCCCTCCGTTTAGAGAGGGGTTATGCTAGTTATTTACCGTAAAATCCTGCACTAAA | | | |
| plantSa-F | **GCGAATTAATACGACTCACTATAGGGCTTAAGTATAAGGAGGAAAAAAT**ATGTATAATAGGTTAGCCGGTCGTATCGGT | plantSα | plantaricin Sα LP | |
| plantSa-R | AAACCCCTCCGTTTAGAGAGGGGTTATGCTAGTTAAATTAAGAATAAACCTGCAACCGC | | | |
| plantSb-F | **GCGAATTAATACGACTCACTATAGGGCTTAAGTATAAGGAGGAAAAAAT**ATGAAACACGGTTTGGGATACCACATTGTT | plantSβ | plantaricin Sβ LP | |
| plantSb-R | AAACCCCTCCGTTTAGAGAGGGGTTATGCTAGTTAAATTGCATTAAGAAAACCTTCACC | | | |
| lafA-F | **GCGAATTAATACGACTCACTATAGGGCTTAAGTATAAGGAGGAAAAAAT**ATGAAAGGTAGTAAAGGGAGCTCTTATGTG | lafA | lactacinF lafA LP | |
| lafA-R | AAACCCCTCCGTTTAGAGAGGGGTTATGCTAGTTAAAAACCTCCTGTCGCACCTGTAAC | | | |

| | | | | |
|---|---|---|---|---|
| sapro-F | **GCGAATTAATACGACTCACTATAGGGCTTAAGTATAAGGAGGAAAAAAT**ATGGGAGCTTTTTTG AAATTTGTTGGA | sapro | saprophyticin S | *S. saprophyticus* P1CEA4 |
| sapro-R | <u>AAACCCCTCCGTTTAGAGAGGGGTTATGCTAGTTA</u>TTATGTCCAAAGTTTTTTAATCTG | | | |

[a] The T7 promoter sequence is shown in bold, and the T7 transcription terminator is underlined.

[b] All primers contain the T7 promoter or the T7 transcription terminator and part of the mature sequence of the predicted bacteriocins.

LP, refers to like-peptide.

**Table S3**. Bacteriocins identified, names, and amino acid sequence of bacteriocins encoded by the most active Gram-negative strains identified in this study.

| **Identification** | **Strain** | **Bacteriocin** | **Amino acid sequence** |
|---|---|---|---|
| *Pseudomonas alcaligenes* | PG7 | S-type pyocin | MTLLRRIDMSGYVANNRDVRSAPEIPVYNGAFDQQPRRQTDRPSPLMPEPLPVPPTQCVFAKPNSLPLGSLDYPSVVPAELASAYGQTAILA<br>TTDVPAAGGGLLLARASGQLVGGGTWAIQSAAGAGGTAAGSGATGAAGSGILATAATTAIGFVALLWPSPMGSSDLYPKSELEVLSTAKT<br>RLRFHVEHDWVNGSIRTYGFHTSSRSGFDSVPVVAARAQGEQAVVDLGDGVTILWTPQVDPAVGAPPPPEDIQGLTETVWIYPVSQNAAQ<br>ALENPIYPSDYKDFIITFPDHPGVQPVYVVLSTQLEKNKVRGREFEDEVYGDYSSTRSETGREVTVKTKSGTRTRIDMVGREPDGTISCVECKS<br>SDTAPLTPNQKVAFPEIEESGAVVVGKGKPGFPGGTEIPPTKVDVVRPN |
| *Escherichia coli* | PG9 | Bacteriocin | MGYGLLDIANQSRREALQGISDADRRREEIEAANKQMAAQQKAQNKQNIGTGIGTGAAIGASVGGPVGAVAGAVIGGIAGSLF |
| | | Colicin E6 | MSGGDGRGHNTGAHSTSGNINGGPTGLGVGGGASDGSGWSSENNPWGGGSGSGIHWGGGSGHGNGGGNGNSGGGSGTGGNLSAVAA<br>PVAFGFPALSTPGAGGLAVSISAGALSAAIADIMAALKGPFKFGLWGVALYGVLPSQIAKDDPNMMSKIVTSLPADDITESPVSSLPLDKAT<br>VNVNVRVVDDVKDERQNISVVSGVPMSVPVVDAKPTERPGVFTASIPGAPVLNISVNNSTPAVQTLSPGVTNNTDKDVRPAGFTQGGNTR<br>DAVIRFPKDSGHNAVYVSVSDVLSPDQVKQRQDEENRRQQEWDATHPVEAAERNYERARAELNQANEDVARNQERQAKAVQVYNSRK<br>SELDAANKTLADAIAEIKQFNRFAHDPMAGGHRMWQMAGLKAQRAQTDVNNKQAAFDAAAKEKSDADAALSSAMESRKKKEDKKR<br>NAENKLNEEKNKPRKGVKDYGHDYHPDPKTEDIKGLGELKEGKPKTPKQGGGGKRARWYGDKGRKIYEWDSQHGELEGYRASDGQHL<br>GSFEPKTGNQL |
| | | Colicin Ia | ---<br>REARSLIEQAEKRQKDAQNADKKAADMLAEYERRKGILDTRLSELEKNGGAALAVLDAQQARLLGQQTRNDRAISEARNKLSSVTESLKT<br>ARNALTRAEQQLTQQKNTPDGKTIVSPEKFPG<br>RSSTNHSIVVSGDPRFAGTIKITTSAVIDNRANLNYLLTHSGLDYKRNILNDRNPVVTEDVEGDKKIYNAEVAEWDKLRQRLLDARNKITSA<br>ESAINSARNNVSARTNEQKHANDALNALLKEKENIRSQLADINQKIAEEKRKRDEINMVKDAIKLTSDFYRTIYDEFGKQASELAKELASVS<br>QGKQIKSVDDALNAFDKFRNNLNKKYNIQDRMAISKALEAINQVHMAENFKLFSKAFGFTGKVIDRYDVAVELQKAVKTDNWRPFFVKL<br>ESLAAGRAASAVTAWAFSVMLGTPVGILGFAIIMAAVSALVNDKFIEQVNKLIGI |
| | | Colicin E1 | MEEKQKQVTASETRLNQISSEINGIQKAISQANNKRSTAVSRIHDAEDNLKIAQTNLLNSQIKDAVDATVSFYQTLSEKYGEKYSKMAQELA<br>DKSKGKKISNVNEALAAFEKYKDVLNKKFSKADRDAIFNALESVKYEDWAKHLDQFAKYLKITGHVSFGYDVVSDILKIKDTGDWKPLFLT<br>LEKKAVDAGVSYVVVLLFSVLAGTTLGIWGIAIVTGILCAFIDKNKLNTINEVLGI |
| *Escherichia coli* | PG14 | Microcin H47 | MGFEGFWSGQAGKTEVKYDGVYMREITESQLRYISGAGGAPATSANAAGAAAIVGALAGIPGGPLGVVVGAVSAGLTTAIGSTVGSGSASS<br>SAGGGS |
| | | Microcin M | MRKLSENEIKQISGGDGNDGQAELIAIGSLAGTFISPGFGSIAGAYIGDKVHSWATTATVSPSMSPSGIGLSSQFGSGRGTSSASSSAGSGS |
| | | Colicin Ia | MSDPVRITNPGAESLGYDSDGHEIMAVDIYVNPPRVDVFHGTPPAWSSFGNKTIWGGNEWVDDSPTRSDIEKRDKEITAYKMTLSAQQKE<br>NENKRTEAAKRLSAAIAAREKDENTLKTLRAGNADAADITRQEFRLLQAELREYGFRTEIAGYDALRLHTESRMLFADADSLRISPREARSLI<br>EQAEKRQKDAQNADKKAADMLAEYERRKGILDTRLSELEKNGGAALAVLDAQQARLLGQQTRNDRAISEARNKLSSVTESLNTARNALT<br>RAEQQLTQQKNTLDGKTIVSPEKFPGRSSTNHSIVVSGDPRFAGTIKITTSAVIDNRANLNYLLTHSGLDYKRNILNDRNPVVTEDVEGDKKI<br>YNAEVAEWDKLRQRLLDARNKITSAESAVNSARNNLSARTNEQKHANDALNALLKEKENIRNQLAGINQKIAEEKRKQDELKATKDAIN<br>FTTEFLKSVSEKYGAKAEQLAREMAGQAKGKKIRNVEEALKTYEKYRADINKKINAKDRAAIAAALESVKLSDISSNLNRFSRGLGYAGKFT<br>SLADWITEFGKGVRTENWRPLFVKTEAIIAGNAATALVALVFSILTGSALGIIGYGLLMAVTGALIDESLVEKANKFWGI |
| | | Microcin V | MRTLTLNELDSVSGGASGRDIAMAIGTLSGQFVAGGIGAAAGGVAGGAIYDYASTHKPNPAMSPSGLGGTIKQKPEGIPSEAWNYAAGRL<br>CNWSPNNLSDVCL |
| *Escherichia coli* | PG15 | Microcin H47 | MGFEGFWSGQAGKTEVKYDGVYMREITESQLRYISGAGGAPATSANAAGAAAIVGALAGIPGGPLGVVVGAVSAGLTTAIGSTVGSGSASS<br>SAGGGS |
| | | Microcin M | MRKLSENEIKQISGGDGNDGQAELIAIGSLAGTFISPGFGSIAGAYIGDKVHSWATTATVSPSMSPSGIGLSSQFGSGRGTSSASSSAGSGS |

| | | | |
|---|---|---|---|
| | | Colicin Ia | MSDPVRITNPGAESLGYDSDGHEIMAVDIYVNPPRVDVFHGTPPAWSSFGNKTIWGGNEWVDDSPTRSDIEKRDKEITAYKMTLSAQQKE<br>NENKRTEAAKRLSAAIAAREKDENTLKTLRAGNADAADITRQEFRLLQAELREYGFRTEIAGYDALRLHTESRMLFADADSLRISPREARSLI<br>EQAEKRQKDAQNADKKAADMLAEYERRKGILDTRLSELEKNGGAALAVLDAQQARLLGQQTRNDRAISEARNKLSSVTESLNTARNALT<br>RAEQQLTQQKNTLDGKTIVSPEKFPGRSSTNHSIVVSGDPRFAGTIKITTSAVIDNRANLNYLLTHSGLDYKRNILNDRNPVVTEDVEGDKKI<br>YNAEVAEWDKLRQRLLDARNKITSAESAVNSARNNLSARTNEQKHANDALNALLKEKENIRNQLAGINQKIAEEKRKQDELKATKDAIN<br>FTTEFLKSVSEKYGAKAEQLAREMAGQAKGKKIRNVEEALKTYEKYRADINKKINAKDRAAIAAALESVKLSDISSNLNRFSRGLGYAGKFT<br>SLADWITEFGKGVRTENWRPLFVKTEAIIAGNAATALVALVFSILTGSALGIIGYGLLMAVTGALIDESLVEKANKFWGI |
| | | Microcin V | MRTLTLNELDSVSGGASGRDIAMAIGTLSGQFVAGGIGAAAGGVAGGAIYDYASTHKPNPAMSPSGLGGTIKQKPEGIPSEAWNYAAGRL<br>CNWSPNNLSDVCL |
| *Escherichia coli* | PG18 | Colicin Ib | MSDPVRITNPGAESLGYDSDGHEIMAVDIYVNPPRVDVFHGTPPAWSSFGNKTIWGGNEWVDDSPTRSDIEKRDKEITAYKNTLSAQQKEN<br>ENKRTEAGKRLSAAIAAREKDENTLKTLRAGNADAADITRQEFRLLQAELREYGFRTEIAGYDALRLHTESRMLFADADSLRISPREARSLIE<br>QAEKRQKDAQNADKKAADMLAEYERRKGILDTRLSELEKNGGAALAVLDAQQARLLGQQTRNDRAISEARNKLSSVTESLKTARNALTR<br>AEQQLTQQKNTPDGKTIVSPEKFPGRSSTNHSIVVSGDPRFAGTIKITTSAVIDNRANLNYLLTHSGLDYKRNILNDRNPVVTEDVEGDKKIY<br>NAEVAEWDKLRQRLLDARNKITSAESAINSARNNVSARTNEQKHANDALNALLKEKENIRSQLADINQKIAEEKRKRDEINMVKDAIKLT<br>SDFYRTIYDEFGKQASELAKELASVSQGKQIKSVDDALNAFDKFRNNLNKKYNIQDRMAISKALEAINQVHMAENFKLFSKAFGFTGKVID<br>RYDVAVELQKAVKTDNWRPFFVKLESLAAGRAASAVTAWAFSVMLGTPVGILGFAIIMAAVSALVNDKFIEQVNKLIGI |
| *Escherichia coli* | P8CEA3 | Microcin V | MSPSGLGGTIKQKPEGIPSEAWNYAAGRLCNWSPNNLSDVCL |
| | | Microcin B17 | MELKASEFGVVLSVDALKLSRQSPLGVGIGGGGGGGGGGSCGGQGGGCGGCSNGCSGGNGGSGGSGSHI |
| | | Colicin E1 | METAVAYYKDGVPYDDKGQVIITLLNGTPDGSGSGGGGGKGGSKSESSAAIHATAKWSTAQLKKTQAEQAARAKAAAEAQAKAKANRD<br>ALTQRLKDIVNEALRHNASRTPSATELAHANNAAMQAEAERLRLAKAEEKARKEAEAAEKAFQEAEQRRKEIEREKAETERQLKLAEAEE<br>KRLAALSEEAKAVEIAQKKLSAAQSEVVKMDGEIKTLNSRLSSSIHARDAEMKTLAGKRNELAQASAKYKELDELVKKLSPRANDPLQNRP<br>FFEATRRRVGAGKIREEKQKQVTASETRINRINADITQIQKAISQVSNNRNAGIARVHEAEENLKKAQNNLLNSQIKDAVDATVSFYQTLTE<br>KYGEKYSKMAQELADKSKGKKIGNVNEALAAFEKYKDVLNKKFSKADRDAIFNALASVKYDDWAKHLDQFAKYLKITGHVSFGYDVVS<br>DILKIKDTGDWKPLFLTLEKKAADAGVSYVVALLFSLLAGTTLGIWGIAIVTGILCSYIDKNKLNTINEVLGI |
| *Escherichia coli* | P8COA2 | Bacteriocin | MGYGLLDIANQSRREALQGISDADRRREEIEAANKQMAAQQKAQNKQNIGTGIGTGAAIGASVGGPVGAVAGAVIGGIAGSLF |
| | | Colicin Ib | MSDPVRITNPGAESLGYDSDGHEIMAVDIYVNPPRVDVFHGTPPAWSSFGNKTIWGGNEWVDDSPTRSDIEKRDKEITAYKNTLSVQQKEN<br>ENKRTEAGKRLSAAIAAREKDENTLKTLRAGNADVADITRQEFRLLQAELREYGFRTEIAGYDALRLHTESRMLFADADSLRISPREARSLIE<br>QAEKRQKDAQNADKKAADMLAEYERRKGILDTRLSELEKNGGAALAVLDAQQARLLGQQTRNDRAISEARNKLSSVTESLKTARNALTR<br>AEQQLTQQKNTPDGKTIVSPEKFPGRSSTNHSIVVSGDPRFAGTIKITTSAVIDNRANLNYLLTHSGLDYKRNILNDRNPVVTEDVEGDKKIY<br>NAEVAEWDKLRQRLLDARNKITSAESAVNSARNNVSARTNEQKHANDALNALLKEKENIRSQLADINQKIAEEKRKRDEINMIKDAIKLT<br>SDFYRTIYDEFGKQASELAKELASVSQGKQIKSVDDALNAFDKFRNNLNKKYSIQDRMAISKALEAINQVHMAENFKLFSKAFGFTGKVIDR<br>YDVAVELQKAVKTDNWRPFFVKLESLAAGRAASAVTAWTFSVMLGTPVGILGFAIIMAAVSAFVNDKFIEQVNKLIGI |
| | | Colicin S4 | MAKELSGYGPTAGESVGGTGANLNQQGGNNNSNSGVHWGGGSGSGNGGGEHGSQTGWGWSKTNNPDVPPYVDDNGQVRITITNGLV<br>KTPVYGVPGAGGNSDVQGGYIPENPNDEVARKWDKNNLPREIDVSIDGFKYRVTLNDNGRAIGILRTGVRPYVGSEKAKAGIMEKINHKT<br>PEEIYEALGFNKDESQRQEKAKQQAEDAWDNLPPNVRKFDVDVEQFHYLVVLDDYGNVLSVTRTGVRPYVGSEKAKAGIMDKVDHKTPE<br>EIYEALGFNNEEPQRQNQAKKAAYDVFYSFSMNRDRIQSDILNKSAEVISDIGNKVGDYLGDAYKSLAREIADDVKNFQGKTIRSYDDAMA<br>SLNKVLSNPGFKLNRADSDALANAWRSIDAQDMANKLGNFSKAFKFADVVMKVEKVREKSIEGYETGNWGPLMLEVESWVLSGIASAVA<br>LGVFSATLGAYALSLGAPAIAVGIVGILLAAVVGALIDDKFADALNNEIIKPAH |
| | | Colicin 10 | ---<br>FPGWRDVQKKLERQLQDKKNEYSSVTNALNSAVSIRDAKKTDVQNAEIKLKEAKDALEKSQVKDSVDTMVGFYQYITEQYGEKYSRIAQD<br>LAEKAKGSKFSSVDEALAAFEKYKNVLDKKISK<br>VERDAIFNAFESVNYDEWAKHLEQISKSLKVTGYLSSAYDVWTDTRKGMETGNWRPLFVTLEKMAVDGGLSRIIVFMFSFIVGVPLGFWGM<br>AIITGIVSAYIGKEELEKLNKLLGI |

| | |
|---|---|
| Colicin E2 | MAALKGPFKFGLWGVALYGVLPSQIAKDDPNMMSKIVTSLPADDITESPVSSLPLDKATVNVNVRVVDDVKDERQNISVVSGVPMSVPVV DAKPTERPGVFTASIPGAPVLNISVNNSTPAVQTLSPGVTNNTDKDVRPAGFTQGGNTRDAVIRFPKDSGHNAVYVSVSDVLSPDQVKQRQ DEENRRQQEWDATHPVEAAERNYERARAELNQANEDVARNQERQAKAVQVYNSRKSELDAANKTLADAIAEIKQFDRFAHDPMSGGH RMWQMAGLKAQRAQTDVNNKQAAFDAAAKEKSDADAALSAAQERRKQKENKEKDAKDKLDKESKRNKPGKATGKGKPVGDKWLD DAGKDSGAPIPDRIADKLRDKEFKNFDDFRRKFWEEVSKDPELSKQFNPGNKKRLSQGLAPRARNKDTVGGRRSFELHHDKPISQDGGVY DMDNLRITTPKRHIDIHRGQ |

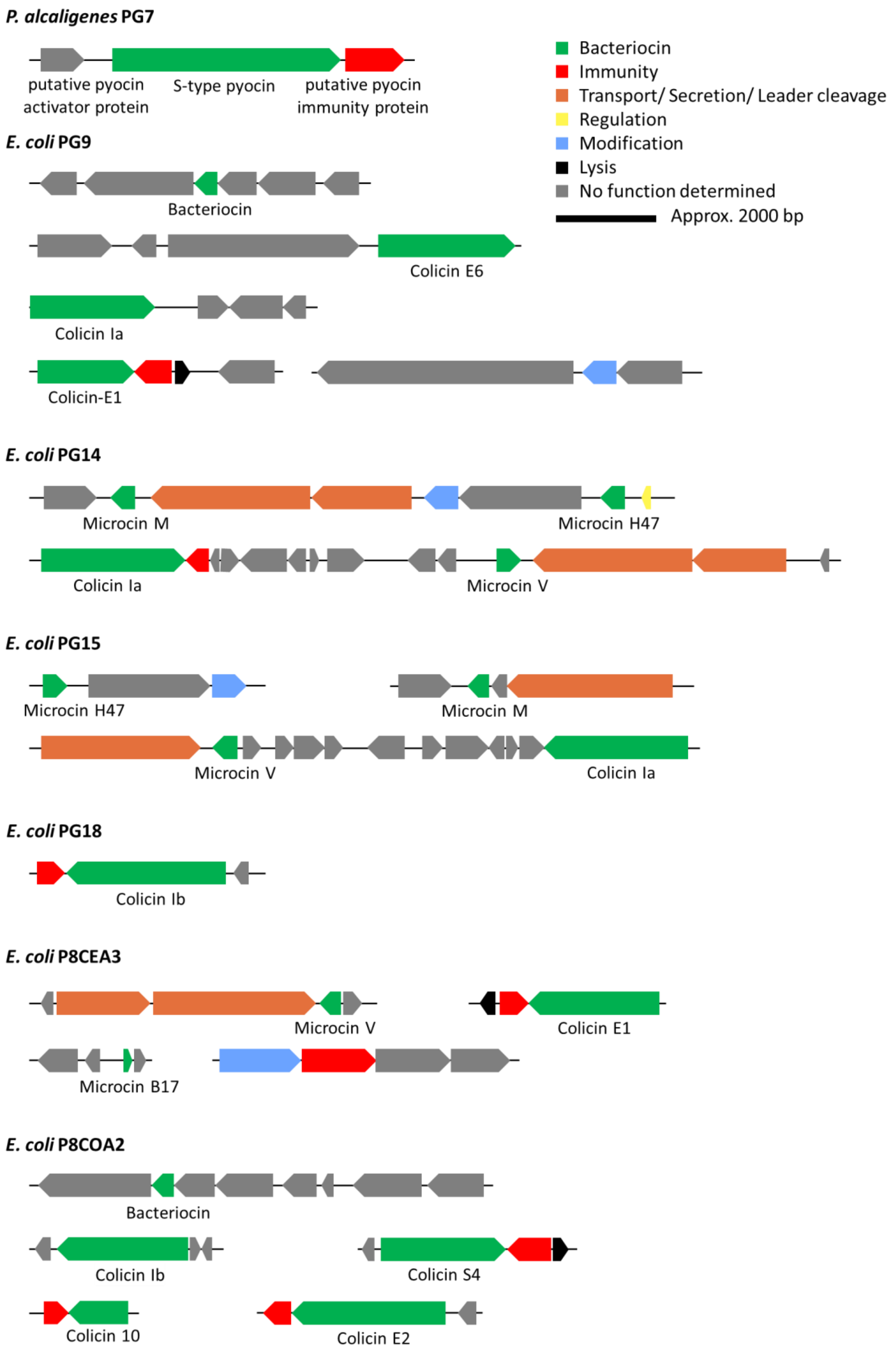


**Figure S1**. Bacteriocin gene clusters (BGC) in genomes of the selected Gram-negative isolates evaluated in this study. ORFs are indicated by arrows and those with a predicted function are indicated by gene identity and/or color.

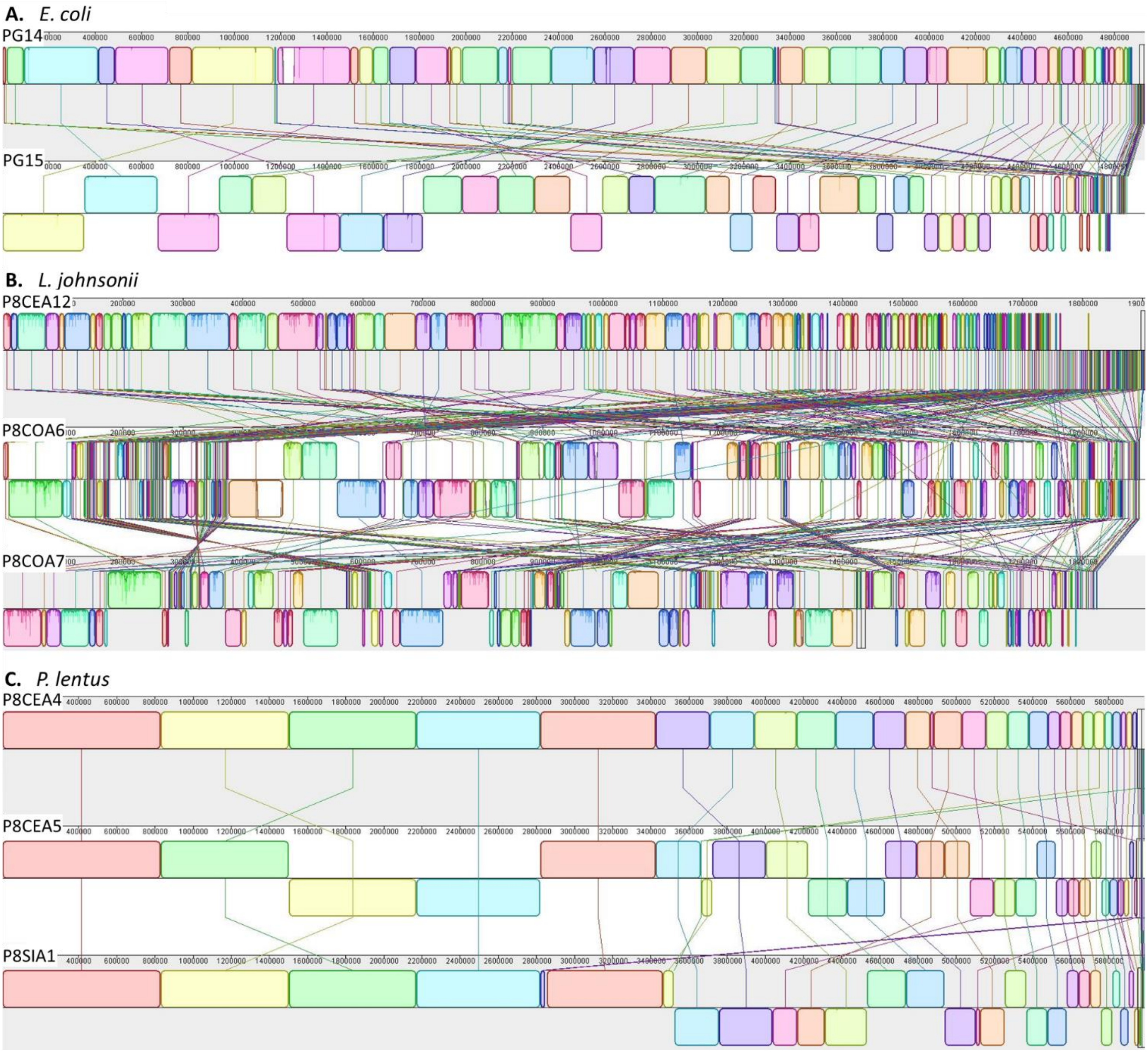


**Figure S2**. Genome alignment of the selected Gram-negative and Gram-positive strains by using the Mauve Multiple Genome Alignment software. (A) *E. coli* PG14 and PG15; (B) *L. johnsonii* P8CEA12, P8COA6 and P8COA7; and (C) *P. lentus* P8CEA4, P8CEA5 and P8SIA1. The alignment is organized into horizontal panels, each representing an input genome sequence. Colored blocks highlight regions of the genome sequence aligned with another genome, suggesting homology and the absence of internal genomic rearrangements. In the first genome, colored blocks are connected by lines to similarly colored blocks in the second and third genomes, showing homologous regions across the genomes. White areas indicate regions that were not aligned and likely contain sequence elements specific to a particular genome. Blocks above the center line represent regions aligned in the forward orientation relative to the first genome sequence, while blocks below the center line represent regions aligned in the inverse complementary orientation.

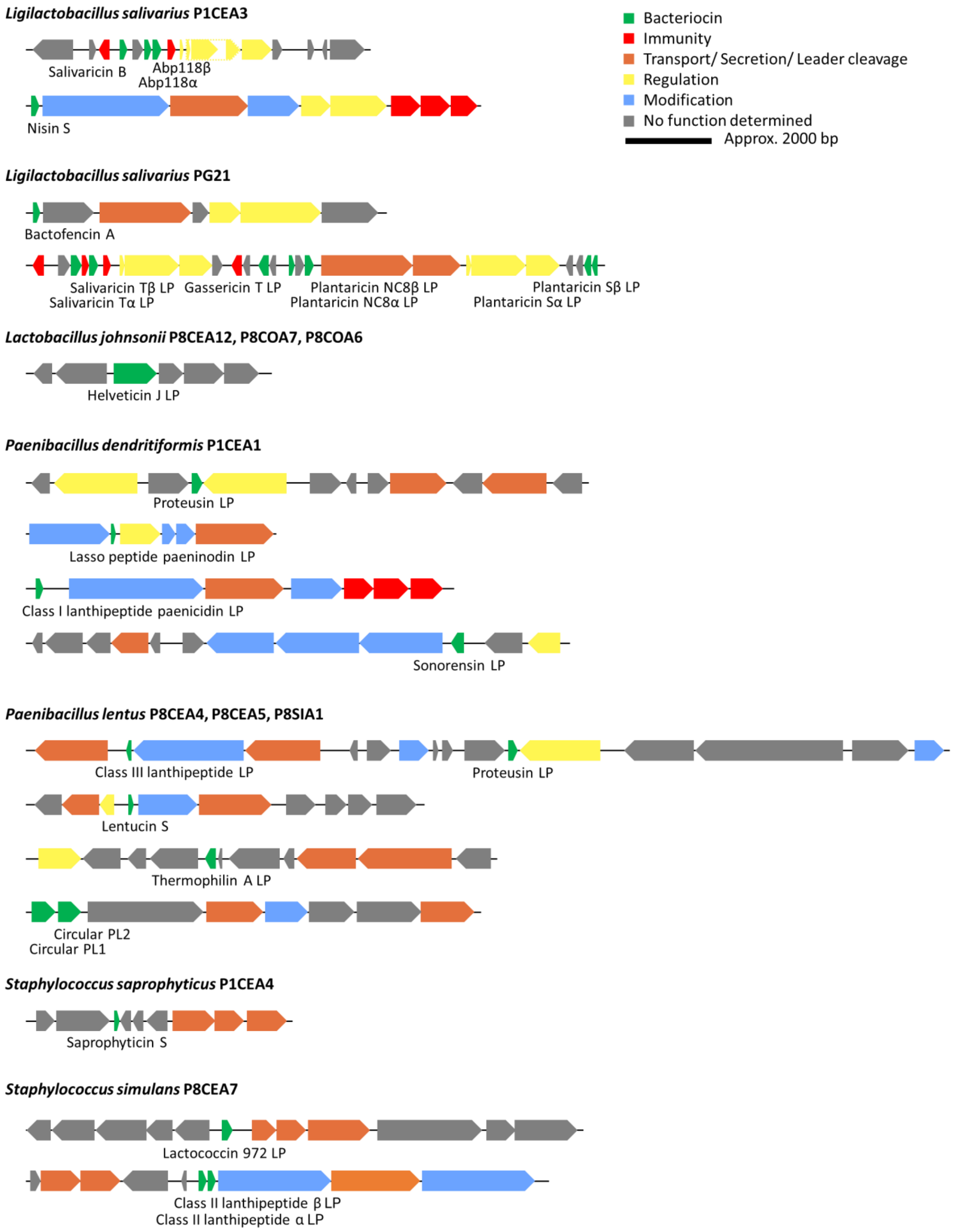


**Figure S3**. Bacteriocin gene clusters (BGC) in genomes of the Gram-positive isolates evaluated in this study. ORFs are indicated by arrows and those with a predicted function are indicated by gene identity and/or color.

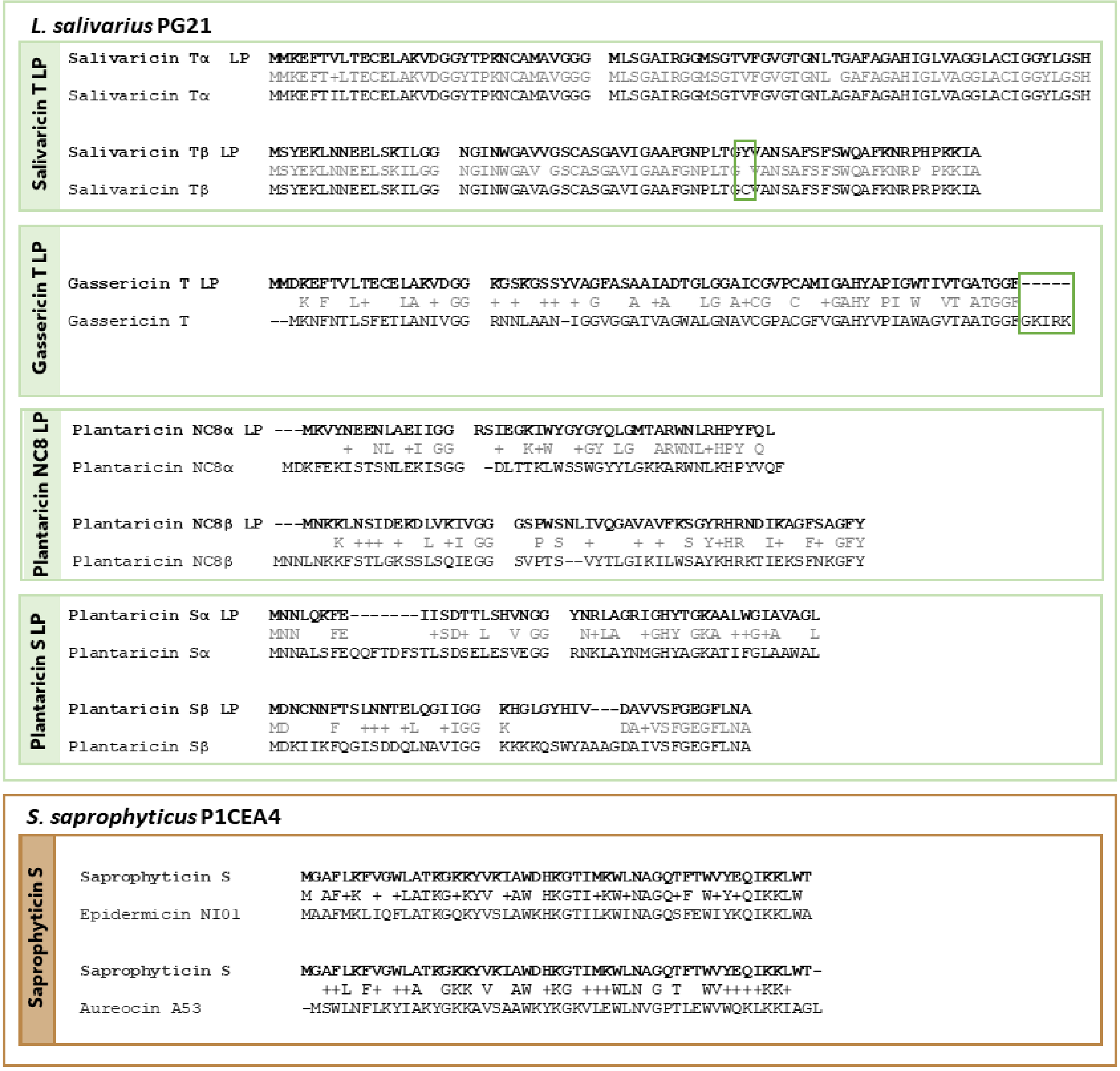


**Figure S4**. Comparison of amino acid sequences of identified class II bacteriocins encoded by *L. salivarius* PG21 and *S. saprophyticus* P1CEA4, as related to the most similar in their class.

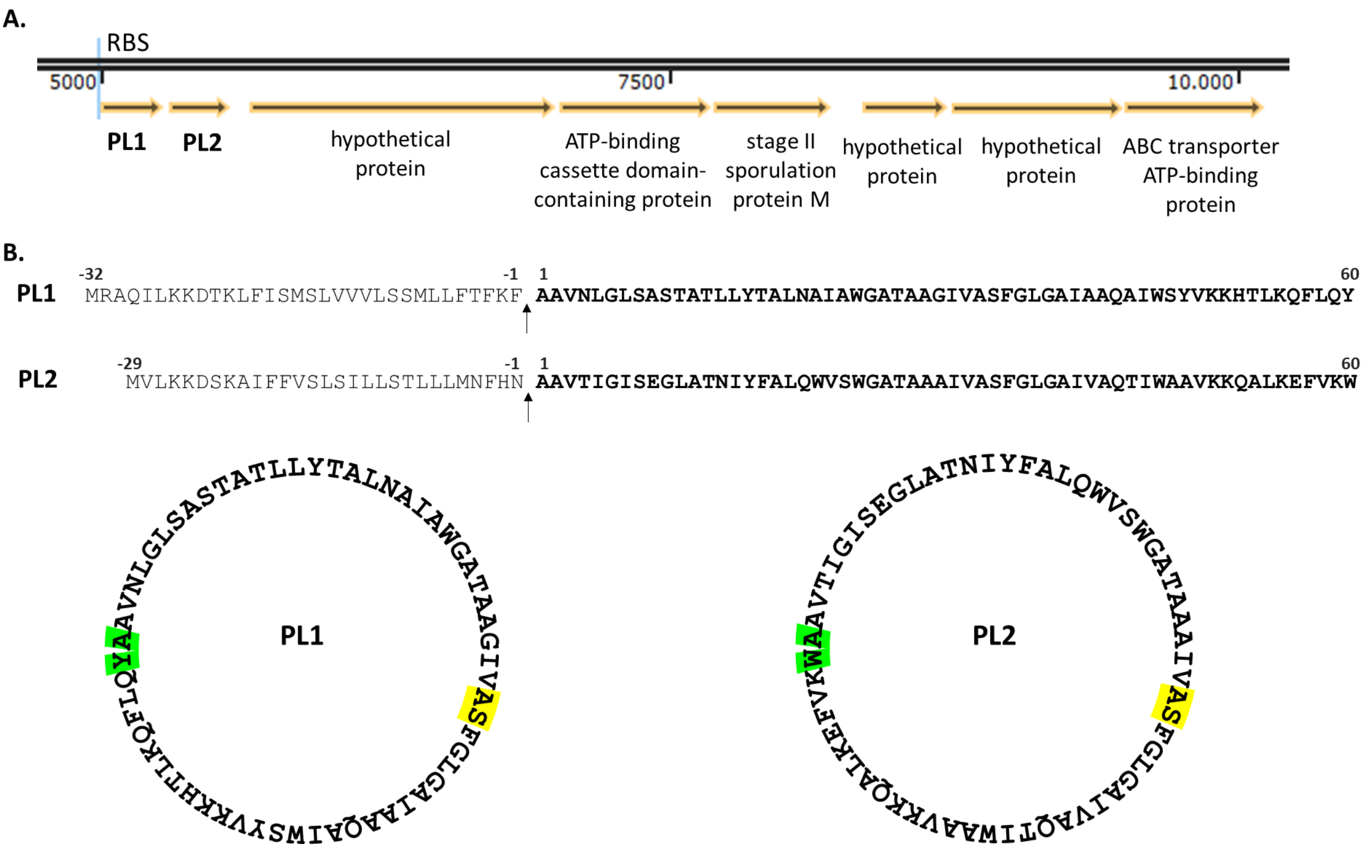


**Figure S5**. Putative circular bacteriocins of the circularin A/uberolysin family identified in the *P. lentus* P8CEA5 genome. (A) Representation of the BGC using SnapGene; RBS in blue; putative circular bacteriocins PL1 and PL2 and BLASTp proteins associated to ORFs. (B) Primary amino acid sequences of the putative circular bacteriocins PL1 and PL2 with the putative cleavage site of the leader peptide indicated by the vertical arrow, and mature amino acid sequences of the putative circular bacteriocins PL1 and PL2; native circularization sites in green and designed sites for circularization of PL1 and PL2 by the IV-CFPS/SIML procedure in yellow.
